# Health worker experiences of implementing TB infection prevention and control: A qualitative evidence synthesis to inform implementation recommendations

**DOI:** 10.1371/journal.pgph.0000292

**Published:** 2022-07-07

**Authors:** Helene-Mari van der Westhuizen, Jienchi Dorward, Nia Roberts, Trisha Greenhalgh, Rodney Ehrlich, Chris C. Butler, Sarah Tonkin-Crine

**Affiliations:** 1 Nuffield Department of Primary Care Health Sciences, University of Oxford, Oxford, United Kingdom; 2 Centre for the AIDS Programme of Research in South Africa (CAPRISA), University of KwaZulu-Natal, Durban, South Africa; 3 Department of Public Health and Family Medicine, University of Cape Town, Cape Town, South Africa; 4 National Institute for Health Research Health Protection Research Unit (NIHR HPRU) in Healthcare Associated Infections and Antimicrobial Resistance, University of Oxford, Oxford, United Kingdom; Corporacion Universitaria Remington, COLOMBIA

## Abstract

Implementation of TB infection prevention and control (IPC) measures in health facilities is frequently inadequate, despite nosocomial TB transmission to patients and health workers causing harm. We aimed to review qualitative evidence of the complexity associated with implementing TB IPC, to help guide the development of TB IPC implementation plans. We undertook a qualitative evidence synthesis of studies that used qualitative methods to explore the experiences of health workers implementing TB IPC in health facilities. We searched eight databases in November 2021, complemented by citation tracking. Two reviewers screened titles and abstracts and reviewed full texts of potentially eligible papers. We used the Critical Appraisals Skills Programme checklist for quality appraisal, thematic synthesis to identify key findings and the GRADE-CERQual method to appraise the certainty of review findings. The review protocol was pre-registered on PROSPERO, ID CRD42020165314. We screened 1062 titles and abstracts and reviewed 102 full texts, with 37 studies included in the synthesis. We developed 10 key findings, five of which we had high confidence in. We describe several components of TB IPC as a complex intervention. Health workers were influenced by their personal occupational TB risk perceptions when deciding whether to implement TB IPC and neglected the contribution of TB IPC to patient safety. Health workers and researchers expressed multiple uncertainties (for example the duration of infectiousness of people with TB), assumptions and misconceptions about what constitutes effective TB IPC, including focussing TB IPC on patients known with TB on treatment who pose a small risk of transmission. Instead, TB IPC resources should target high risk areas for transmission (crowded, poorly ventilated spaces). Furthermore, TB IPC implementation plans should support health workers to translate TB IPC guidelines to local contexts, including how to navigate unintended stigma caused by IPC, and using limited IPC resources effectively.

## Introduction

Globally, *Mycobacterium tuberculosis* (TB) leads to 1.4 million deaths per year [1]. In high TB burden countries, health facilities may become hot spots for TB transmission due to poor TB infection prevention and control (IPC) implementation [2]. The most visible consequence has been health workers falling ill with TB at an incidence rate 2.94 higher than the general population [3]. Nosocomial TB spread in health facilities also places patients at risk, although this phenomenon has been more difficult to quantify.

At the start of the COVID-19 pandemic, exisiting research about TB IPC implementation was used to inform IPC strategies for COVID-19 [4]. Yet despite this forewarning, many of the implementation deficiencies that have long plagued TB IPC programmes became apparent on a global scale during the COVID-19 pandemic: the difficulty of identifying patients who have not yet been diagnosed but pose a risk of infection to others; health facilities without adequate ventilation infrastructure; unreliable supply chains of particulate filter respirators; health worker discomfort when using respirators for prolonged periods; social influences on the acceptability of mask wearing and the negative effect of unclear or changing guidelines [4–7]. Nosocomial transmission of the SARS-CoV-2 virus has, similarly to TB, played a significant role in the spread of the COVID-19 pandemic [8]. The difficulties in preventing transmission of old and new pathogens have exposed the complexity associated with implementing airborne IPC measures in health facilities, and raised the question of potential synergy in reducing nosocomial transmission of different respiratory infectious diseases.

Yet, within the TB IPC field, poor TB IPC is most commonly attributed to a failure on an invidual health worker level to implement TB IPC guidelines, with researchers often recommending further TB IPC training to remedy this [9]. This framing ignores the complexity associated with TB IPC as intervention, and does not take implementation difficulties into consideration. The 2019 World Health Organisation (WHO) updated TB IPC guidelines have responded to this by discussing implementation challenges of each TB IPC subcomponent (administrative controls, environmental controls, and personal protective equipment) [10]. The implementation considerations were based on the expert opinion of the guideline committee.

In contrast to using expert opinion, synthesising qualitative research is a more systematic method of identifying implementation considerations to accompany guidelines [11]. A qualitative evidence synthesis allows the exploration of the complexity within the health system and the complexity inherent in the intervention [12]. In addition to this, GRADE-CerQUAL (Grading of Recommendations, Assessment, Development and Evaluation and Confidence in the Evidence from Reviews of Qualitative research) methods allow researchers to express confidence in the qualitative research recommendations in a transparent way [13].

Exisiting reviews on TB IPC have not addressed this: two mixed method reviews of TB IPC implementation led by Tan [9] and Zwama [14] focused on identifying research gaps and neither used qualitative evidence synthesis. Tan and colleagues’ 2020 systematic review explored the barriers to and facilitators of TB IPC in low- and middle-income countries from the perspective of healthcare workers [9]. They used an existing macro, meso, and micro health systems framework deductively to classify these barriers and facilitators, with the authors concluding that a major limitation of existing research is ‘framing the problem as one of poor adherence to guidelines by healthcare workers’ [9]. Zwama and collegues’ 2021 scoping review looked at health system factors influencing TB IPC implementation [14]. Using a framework developed by Sheikh, TB IPC implementation considerations were categorised into political context, policy decisions, system hardware and system software. The authors proposed a greater focus on the complexity associated with TB IPC using a whole systems approach [14].

We approached this topic by paying particular attention to descriptions of complexity—looking for ‘adaptive solutions’ or workarounds that heath workers use when resources are limited, and how they prioritise different components of TB IPC as intervention [15]. We aimed to use an inductive approach to review qualitative evidence of the complexity associated with implementing TB IPC, in order to guide the development of TB IPC implementation plans.

## Methods

This review used Qualitative Evidence Synthesis (QES) methods for developing implementation recommendations [16]. The protocol for this review was preregistered on PROSPERO. (ID CRD42020165314) We made two changes to the pre-registered protocol by removing search restrictions for dates and high TB burden countries as we decided to broaden the scope of this review.

### Research question

Our initial research question—what are the experiences of health workers implementing TB IPC?—was used as starting point to develop follow-on research questions informed by provisional data analysis. These were:

- What are the main narratives in health worker explanations of why TB IPC is difficult to implement?
- What are the underlying assumptions that health workers and researchers make about implementing TB IPC, that need a critical assessment?
- How can exploration of the complexity of TB IPC as intervention, and the complexity of the system in which it is introduced, be used to support implementation plans?

### Criteria for considering studies for this review

We included primary research studies using qualitative methods and analysis, or mixed methods studies with a qualitative analysis component where qualitative results were reported separately. Studies had to report an account provided by health workers of some aspect of their experience with implementing TB IPC. The term ‘health workers’ included doctors, nurses, dentists, radiologists, community health workers and other health personnel like administrators who implement TB IPC. Healthcare settings included primary health care facilities, district hospitals, and hospitals providing specialist care or community-based care.

### Search strategy and screening

We searched eight databases including Medline [OvidSP) [1946—present], Embase (OvidSP) [1974—present], Global Health (OvidSP) [1973–2021 Week 46], CINAHL (EBSCOHost) [1982—present], African Index Medicus (via https://www.globalindexmedicus.net/) and Science Citation Index, Social Science Citation Index and Conference Proceedings Citation Index—Science (Web of Science Core Collection) [1900—present] on 23rd November 2021. We developed a search strategy based on title and abstract keywords and subject headings to describe our key concepts of health professionals, TB infection prevention and control and experiences. We applied a filter for qualitative research and did not limit by date or language. (For full search strategies see S1 Appendix). References were exported to Endnote 20 for deduplication prior to screening. On 22 December 2021 we also conducted forward and backward citation tracking of publications meeting the inclusion criteria by looking at references of included studies and studies citing them. Only studies with abstracts that were available in English were screened.

### Selection process

The titles and abstracts of all publications identified in the search were assessed using the inclusion criteria by two reviewers (HvdW and JD or HvdW and STC). Disparities were resolved through discussion with a different third reviewer (JD or STC). Full texts were obtained and screened against the inclusion criteria by two reviewers (HvdW and JD or STC). Reasons for exclusions were documented.

### Quality appraisal

The quality of research papers was independently evaluated by two reviewers (HvdW and JD or STC) using the Critical Appraisals Skills Programme (CASP) checklist [17]. We did not exclude studies based on quality but considered the CASP score when determining the contribution of studies to the key findings of the evidence synthesis and when expressing the confidence in the key findings.

### Reflexivity statement

This review was led by HvdW, a doctoral researcher using qualitative research methods. She has experience of developing and presenting training on TB IPC implementation for South African health workers and working as a health worker in this high TB burden setting. This brings a pragmatic approach to this review, which prioritises how TB IPC implementation can be improved in practice. Other collaborators bring expertise in public health, health worker behaviour change research, occupational health, complexity theory, and the experiences of frontline health workers in a high TB burden country.

### Data analysis

The studies were analysed using thematic synthesis as method. It involved three stages of analysis (led by HvdW): line-by-line coding of the findings and discussion sections of primary studies, organising the codes into related areas to develop descriptive themes, and then developing analytical themes [18]. The descriptive and analytical themes were developed in discussion with all authors. The coding was done using MAXQDA2020 software. Two papers (2/37, ~5%), selected based on the richness of data, were second coded by STC and compared with the coding done by HvdW. This was used to discuss the approach to coding (balancing detail with summarising main themes) and to inform decisions on what would be coded as data. We considered both the original data, as it is reported in the results section of the paper, as well as the original researchers’ interpretation in each study as described in the discussion section.

After completing the initial analysis, we explored the one theme in more detail by looking at descriptions of TB IPC measures that were being implemented outside of what is recommended in international guidelines. We explored how misconceptions (an incorrect understanding), assumptions (inferring a relationship where there may be none), and uncertainty about guidelines influenced TB IPC implementation. We also noted ‘workarounds’ which health workers used when facing resource constraints in implementation. We based the classification of these underlying factors on what we as review authors viewed to be effective TB IPC. While we acknowledge our interpretation may be contested by other infection control practioners, a more extensive discussion of the evidence is beyond the scope of this review. Our aim was to demonstrate the wide variety of TB IPC measures being used, some which are unlikely to be effective in preventing TB spread.

### Appraisal of certainty of review findings

The GRADE-CERQual approach was used to describe our confidence in our review findings. It is based on considering the subcomponents as defined by Lewin and colleagues [13]:

- methodological limitations: ‘concerns about the design or conduct of the primary studies that contributed evidence to an individual review finding’,
- coherence: ‘how clear and cogent the fit is between the data from the primary studies and a review finding that synthesises that data’,
- adequacy of data: ‘an overall determination of the degree of richness and quantity of data supporting a review finding’, and
- relevance: ‘the extent to which the body of evidence from the primary studies supporting a review finding is applicable to the context specified in the review question.’

The CERQual evidence profile was developed by discussing the review findings among authors and selecting the key findings. We developed an implementation consideration accompanying each of the key findings, which is presented in the discussion section. We used guidance developed by Glenton and colleagues to develop the implementation considerations [16].

## Results

### Description of studies

**Fig 1:**
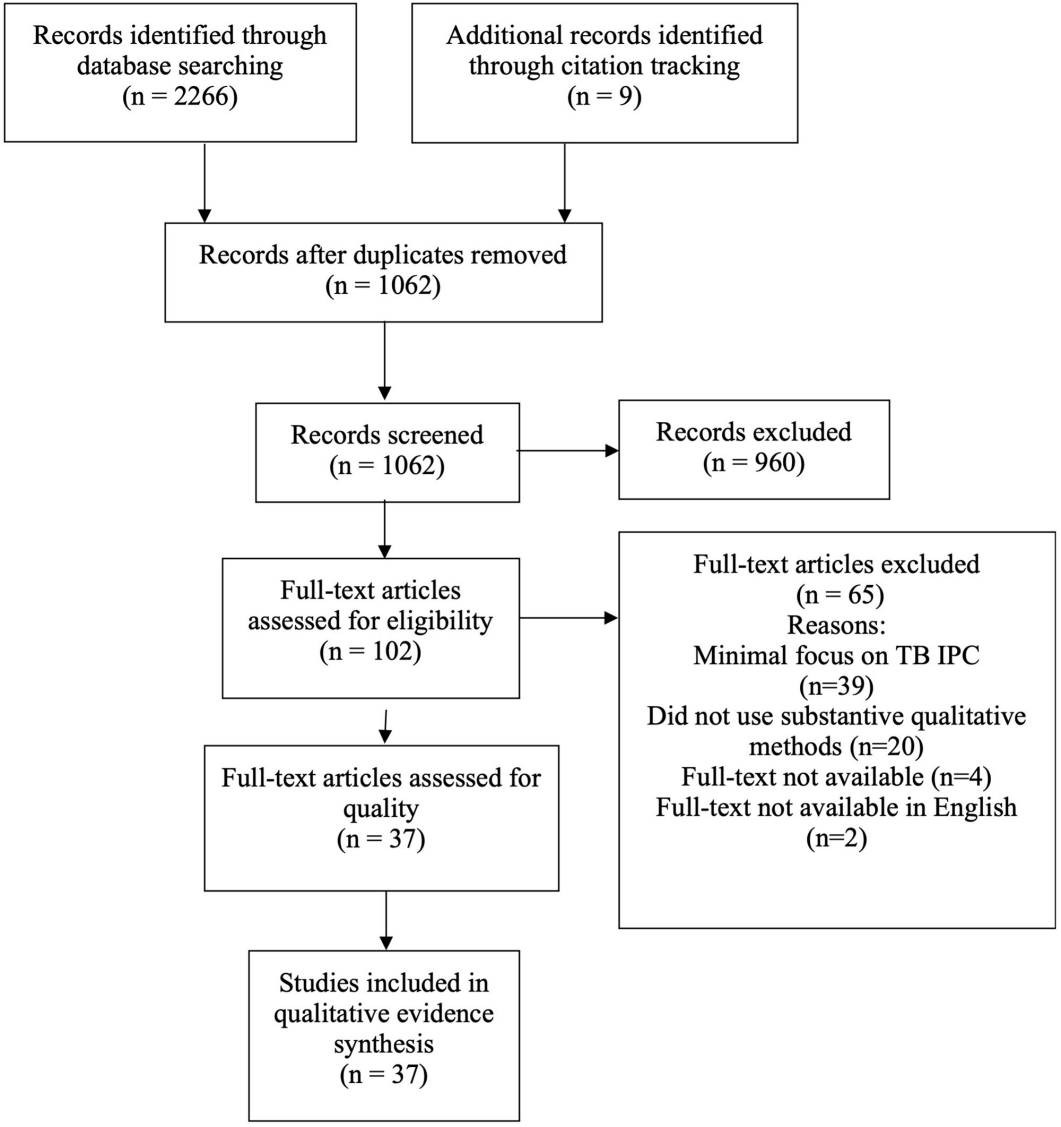
PRISMA flow diagram.

We considered 1 062 titles and abstracts and 102 full text papers for inclusion. A total of 37 studies were included in the synthesis, all of which were published after 2010 (Fig 1).

S1 Table summarises information of the included studies, including key findings and use of theoretical frameworks. S2 Table provides the CASP score of all of the included studies. The research studies were undertaken in fourteen different low or middle incoming countries (based on the World Bank classification [19]): South Africa (n = 16), Nigeria (n = 4), the Dominican Republic (n = 3), India (n = 2), Uganda (n = 2), Ethiopia (n = 2), with one study from Mozambique, Ghana, Papa New Guinea, Brazil, Zambia, Bangladesh, Indonesia and Russia, respectively. All of the countries except the Dominican Republic appear among the 2021 WHO high-burden countries for TB, HIV-associated TB and drug-resistant TB [20]. Africa as region had strong representation, followed by Asia and the Americas. The perspectives of health workers in the qualitative research included in this review are therefore those of workers in low—middle income, high TB burden countries.

The primary research was based at different types of health care facilities, with the majority focussing on TB IPC at hospital-level (n = 12), or for specialised TB or DR-TB units (n = 12), while some were based at primary health care facilities (n = 5) and other studies combined data from different facility types (n = 6). For studies with a rural focus, see for example Marme [21] and Tshitangano [22–24]. Health workers of all groups were included, most commonly nurses, but also doctors, laboratory staff, TB programme staff, housekeeping staff and facility managers. Two studies specifically recruited health workers who had experiences of occupational TB [25, 26].

### Thematic synthesis main findings

Using thematic synthesis, we developed five themes. We were guided by narratives frequently expressed by health workers working in different settings (recurring patterns), and sought to reflect the depth of explanations (rich descriptions). Some studies represented a simplified perspective of the implementation challenges of TB IPC. Other studies approached the subject with greater depth and nuance—providing “complex narratives”. For purposes of contrast, these distinctions are schematically summarised in Table 1.

**Table 1:** Summary of five key themes identified through thematic synthesis.

	Simple narrative	Complex narrative
Theme 1	Frontline health workers receive insufficient training on TB IPC.	Health workers combine TB IPC training with stories of colleagues who have developed TB to inform their perceptions of TB risk. Workplace considerations like cognitive load, organisational culture and workplace safety influence how they weigh these considerations.
Theme 2	TB IPC implementation is impeded by lack of resources, predominantly PPE and health facility infrastructure.	Across differently resourced settings, TB IPC is difficult to implement. This suggests that it is not only the lack of resources that plays a role, but complexity related to the technology (respirators or ventilation systems) and within the health system (e.g., procurement, maintenance, and workflow for infectious patients).
Theme 3	Absent or unclear policies on occupational health and TB IPC and a lack of local implementation plans hinder implementation.	Health workers describe important gaps or contradictions in TB IPC and occupational health policy implementation, e.g., in content (is TB an occupational illness?), in prioritisation (is implementation being funded?), and in local translation of the policy (who is driving it and how does it fit in with existing workflows?).
Theme 4	Patients are unsupportive of TB IPC implementation in health facilities.	Health workers need support to balance their concerns that TB IPC will lead to “stigma” with their ethical responsibility to prevent the spread of TB in health facilities.
Theme 5	Health workers are not following existing TB IPC guidelines, resulting in poor TB IPC implementation.	Health workers have different understandings of how TB spreads, and which TB IPC measures should be prioritised. Existing TB IPC efforts may be failing because of (a) the way that guidelines are interpreted, and (b) which interventions are prioritised.

In the next sections, we discuss the complex narratives that contribute to each theme in depth, highlighting the ten key findings from this review that could be used to inform TB IPC implementation planning. Regarding our confidence in the ten key findings, five have high confidence, three moderate confidence and two low confidence. Table 2 provides an evidence summary of our 10 key findings. For a complete CERQual evidence profile, see S2 Appendix.

**Table 2:** Summary of ten key findings of qualitative evidence synthesis with CERQual assessment.

Summary of review key findings	Studies contributing to the review finding	CERQual assessment of confidence in the evidence	Explanation of CERQual assessment
(1) Health workers describe their perception of their own TB risk as contributing to the importance they attach to TB IPC (e.g., hearing about other health workers who have developed occupational TB)	[26–37]	High confidence	Data from 12 studies, three with methodological limitations. Studies form six different countries with wide geographic spread.
(2) Health workers describe feeling unvalued and unsafe at work, and that they have limited power over their working conditions. The risk of being exposed to TB is part of this insecurity.	[25, 26, 29, 30, 32, 34, 38–42]	Moderate confidence	Data from 11 studies, two with minor methodological limitations. Studies from four different countries, limited geographic spread.
(3) Health workers may stigmatise patients because of their fear of being exposed to TB and compromise clinical care by avoiding contact, shortening consultations, and restricting patient movement, e.g. through fencing wards and locking patient rooms.	[31, 33, 34, 41, 43–48]	Moderate confidence	Data from 10 studies, six with some methodological limitations. Studies predominantly from countries in Africa, therefore has limited geographic spread.
(4) Health workers describe unavailability of particulate filter respirators as the main barrier to using respiratory protective equipment. Examples were given of doctors having preferential access over nurses or housekeeping staff when stock was limited.	[21, 26–31, 33, 35, 40, 41, 45–52]	High confidence	Data from 19 studies, six with methodological limitations. Studies from nine different countries.
(5) Health workers report working in facilities where the infrastructure was not designed for airborne infection control measures, with waiting areas, consultation rooms and wards lacking natural or mechanical ventilation and having an insufficient number of isolation rooms.	[21, 27–30, 32, 33, 38, 40–42, 44, 46–56]	High confidence	Data from 23 studies, eight with methodological limitations. Studies from twelve different countries.
(6) Health workers express uncertainty about whether TB is an occupational illness and whether they need to prove they were infected at work. This leads to underreporting of occupational TB.	[32, 37, 38, 40]	Low confidence	Data from four studies, one with methodological limitations, all from South Africa.
(7) Health workers perceive patients as feeling stigmatised by TB IPC measures, such as being asked to wear a mask for source control. In general, health workers considered stigma an important impediment to implementing TB IPC measures.	[21, 28, 30–32, 36, 39, 56, 57]	High confidence	Data from nine studies, one study with minor methodological limitations and from seven different countries.
(8) Health workers describe the targeting of TB IPC resources towards clinical areas where patients with known TB or drug resistant TB are seen, including TB wards and DOTS centres. Health workers directly caring for such patients have priority access to personal protective equipment. Housekeeping, administrative staff and community health workers are frequently overlooked, as are health workers providing other types of patient care, despite the pervasiveness of TB risk.	[21, 29, 32–34, 36, 41, 42, 45, 47, 48, 50, 52–56]	High confidence	Data from seventeen studies, five with methodological limitations. Studies from ten different countries.
(9) There is a wide variation in the in the duration that different health workers perceive patients with TB to be infectious.	[32, 43, 50]	Low confidence	Data from three studies with minor methodological limitations. Studies based in two countries.
(10) Health workers describe using a variety of IPC measures aimed at preventing the spread of TB, including contact, droplet and airborne precautions.	[23, 28, 34, 40, 42, 44, 46, 47, 49, 54, 56]	Moderate confidence	Data from eleven studies, six with methodological limitations from six countries.

#### Theme 1: Interactions between TB IPC training, risk perception and experiences of workplace safety

Health workers flagged insufficient TB IPC training as an important contributor to poor TB IPC implementation [21, 26, 27, 30, 32–34, 41, 47–49, 52–55]. They described existing IPC training as targeted towards health workers working in TB wards, Directly Observed Therapy Short-course (DOTS) programmes, or at facility managers [32, 55]. In some instances, having TB training led to TB work being delegated to or reserved for these health workers [21, 48]. Gaps in training included uncertainty about which IPC measures are important, a lack of refresher training after graduation, and difficulties in training new staff when there is high employee turnover [32, 37, 50, 53, 56].

Self-perceived risk of developing TB contributed to the importance health workers attached to implementing TB IPC [25, 26, 29, 30, 32, 34, 38–42]. (Table 2, **Key finding 1**) They described being influenced by the experiences of colleagues who had developed TB [27]; for example, they recalled a medical student who developed drug-induced hepatitis due to TB medication and died [33]. Health workers who had witnessed the suffering of drug-resistant TB patients felt incapable of going through it themselves. One nurse at a drug-resistant TB treatment centre in South Africa mentioned, ‘*All that medications and injections—I’d rather kill myself than go through what those poor patients go through*’ [51]. A contributory fear of health workers was that they would infect family members [27, 28, 35, 45].

Health workers’ response to occupational risk fell into three groups. In the first, they perceived themselves not to be at risk of TB and did not value TB IPC training [29]. Misconceptions contributing to this included: the belief that latent TB infection would be protective against developing TB disease [29], and that BCG vaccination [53], ‘eating well’ before examining patients [35] or taking multivitamins [27] prevented TB infection. A second group believed they were at risk of TB, valued TB IPC training and felt equipped to care for TB patients [34]. A third group were aware of their occupational TB risk and received TB IPC training, but were faced with inadequate resources to implement the preventative practices that they knew were important [29, 36]. This is conceptualised as ‘powerlessness’ by Chapman and colleagues, when discussing their findings:

“*If HCWs perceive that their occupational risk is high and beyond their control*, *irrespective of their use of infection control measures*, *such as in understaffed clinical areas*, *excess workloads*, *or absence of an isolation ward*, *then they may feel powerless in their consistent use of M*. *tuberculosis infection control measures*” [29].

Some health workers felt their TB exposure was part of broader concerns about feeling unvalued and unsafe at work, and perceived themselves to have limited power over their working conditions [25, 26, 29, 30, 32, 34, 38–42] (Table 2, **Key Finding 2**). Health workers phrased this as working in a system that did not care about their health. One primary care nurse in South Africa said “….*nobody cares*, *even if I die*, *it doesn’t matter*” [32]. As an example of feeling uncared for by the health system, health workers mentioned that they did not trust the respirators that they are provided as PPE, because they often fitted poorly, involved counterfeit manufacture or unknown brands, or they received a surgical or cloth mask instead of an N95 respirator [27, 30, 35, 40, 41, 51, 54].

Working in conditions that feel unsafe, and feeling poorly equipped to implement TB IPC, may influence patient care. One nurse working in a TB ward in South Africa described this as: “*I feel like I am putting myself in danger to be honest*. *I am scared to come into contact with those patients because most of them are rude and dangerous*” [41]. In some instances, health workers may stigmatise patients because of their concerns about being exposed to TB, and therefore compromise clinical care. Examples included health workers who avoided contact with TB patients, shortened consultations, and restricted patient movement through placing fencing around wards and locking patient rooms [31, 33, 34, 41, 43–48] (Table 2, **Key finding 3**). Health workers described how fear of being infected with TB leads them to avoid TB work, with some health workers viewed caring for TB patients punishment [43] or work for junior staff [45]. They also described examples where patients were declined intensive care, such as dialysis, or care at a referral hospital because of infection risk [46].

For health workers, working in conditions where they felt unsafe blunted their occupational TB risk perception [36]. One professional nurse decribed this transition as: ‘*I used to be scared when I started but I have gone past that now… yesterday we were so busy here*, *there is no time to get paranoid*’ [37]. This included both a high clinical load [29] and the emotional load association with caring for patients who are very ill. A nurse reflected on this: “*The most painful and traumatizing thing in the TB wards is that patients die in large numbers and it is so stressing to see people dying just before you every day*. *… We do not get even debriefing sessions*. *As a result*, *we feel that we are losing it*, *at times we just can’t cope*” [41].

Kallon and collegues group many of these workplace considerations under organisational culture as framework, and argue that the influence of organisational culture on TB IPC use is underresearched and underappreciated. The researchers conclude that the responsibility of implementing TB IPC is incorrectly shifted to individual workers:

*‘The lack of role modelling*, *the under-supply of masks*, *and the vague and poorly enforced protocols around mask-wearing*, *for example*, *all communicated that wearing a mask was less a collectively held and enforced responsibility and more a matter of personal choice and agency’* [36].

#### Theme 2: Technology and organisational implementation challenges of TB IPC in health facilities

Health workers viewed unavailability of sufficient stock of particulate filter respirators as the main barrier to using respiratory protective equipment [21, 26–31, 33, 35, 36, 40, 41, 45–52]. Doctors or health workers in TB wards or DOTS centres had preferential access over nurses, housekeeping staff [49], or staff in other non-TB sections [28] (Table 2, **Key finding 4**). Other factors that contributed to poor access to respirators included high unit cost, poor supply chain management, lack of prioritisation by managers as reflected, for example in poor supply chain management and health workers being expected to purchase their own protective equipment [31, 49].

Fluctuations in the availability of PPE prevented health workers from developing a habit of wearing a respirator, as well as making them feel that sporadic use wass futile. A TB staff member in Mozambique said: ‘*It should be there all the time*. *Because if the material is there today*, *but finished tomorrow*, *I am not interested*, *I am not protected at all […] do I not contract the bacteria when I don’t have the material*?” [28] Health workers described respirators as difficult to use, “*suffocating… and feels hot*”[37] and interfering with make-up. Respirators caused communication difficulties with patients who spoke a different language or who were impaired by ototoxic drug-resistant TB medication and depended on lip reading [31]. Health workers paradoxically described using respirators less in poorly ventilated settings, where they described greater discomfort with use [36].

Health workers often worked in facilities where the infrastructure was not designed for airborne infection control measures, with waiting areas, consultation rooms and wards that lacked natural or mechanical ventilation and insufficient isolation rooms [21, 27–30, 32, 33, 36, 38, 40, 41, 42, 44, 46–56]. (Table 2, **Key finding 5**) One health worker working in Uganda described the struggle they faced to establish a safer waiting room:

“*When we were conducting the TB infection assessment*, *the ventilation was found to be at 0%*. *… The worst thing is that these HIV patients are seated together with TB patients and some of them are … not yet on treatment*. *We requested to move the clinic to a … better ventilated place out there on the veranda*, *but it was rejected*. *So [name of donor funded project] came in and gave us a tent*, *which didn’t help us much because it came without seats and immediately [after] we put it up*, *it broke down and we didn’t use it*. *So we have remained in the same place up to today*” [44].

Some health workers responded to spatial limitations by moving DOT consultations and sputum sample collection outdoors. In some instances this compromised privacy, but was easy to implement [47]. The ventilation measures described by health workers involved optimising natural ventilation—such as opening windows and shifting consultations outdoors. We did not find descriptions of frequent use of mechanical ventilation, air filtration such as High Efficiency Particulate Air (HEPA) filters or upper-room germicidal ultraviolet disinfection [52].

Health workers in primary care and rural health care facilities described receiving fewer resources and worse TB IPC infrastructure than urban and hospital settings [21, 32]. Yet in our broader dataset we noted that health workers in middle income countries (compared to low income countries) and in tertiary hospitals described similar TB IPC implementation barriers to those of low income or rural hospital settings [30, 50], suggesting that TB IPC is difficult to implement across the whole resource spectrum.

#### Theme 3: Policy and implementation plan gaps relating to TB IPC and occupational health

Health workers and researchers highlighted the following obstacles to implementing TB IPC: the lack of a national IPC policy [45], conflicting national TB IPC guidelines [32, 40] and the absence of a local TB IPC implementation plan for facilities [24, 37] and uncertainty about how TB IPC policies translate to the work of different health workers (for example dentists) [36]. They viewed national TB IPC guidelines as instructions that also provided a mechanism to motivate health workers to comply, for example, through a central government audit [32].

Yet health workers mistrusted whether TB IPC policies adequately protected them. For example, they were uncertain about whether such guidelines described the correct duration of infectiousness of TB patients [32] or how TB IPC policy should translate into daily practice. A health worker from South Africa explained the uncertainty: “*Always wear the N95 mask*, *or conserve them due to expense*? *Separate XDR and MDR TB patients*, *or allow them to mix at times*?*”* [40] Health workers described trying to make individual management decisions but felt there was a need for TB IPC implementation plans to address practical concerns, including around respirator re-use [36].

At one facility, a hospital manager said policy implementation, in contrast to policy availability, was the biggest challenge [40]. Involving health workers in discussing and developing the guidelines of a facility was suggested as way to ‘create a sense of ownership’ [28]. One facility identified a facility-based champion as a way of making TB IPC a priority, yet cautioned it should be balanced with maintaining TB IPC as a collective responsibility [37].

Health workers expressed uncertainty about whether TB was legally defined as an occupational illness. Underreporting obscured the extent to which health workers are affected by occupational TB [32, 37, 38, 40] (Table 2, **Key finding 6**) and influenced risk perceptions of health workers and managers [38]. Several health workers viewed poor TB IPC implementation and occupational TB to be an individual’s problem, instead of a health system’s problem [36, 37]. A health worker in South Africa said: ‘*The thing is with TB and being a health worker*, *should I get it*, *I know it’s going to be my problem*. *I won’t be able to prove that I got it here*. *So I guess if you work here*, *it is at your own risk*. *That’s how I feel*’ [37]. Even among health workers who perceived TB to be an occupational illness, and who were aware that they could qualify for compensation, did not trust the bureaucratic progresses of accessing compensation, with one example provided from South Africa [37].

Health workers also described occupational health service shortcomings: lack of trained staff [38], a shift of the responsibility to implement an occupational health policy to untrained staff [38], lack of confidentiality within staff health clinics or occupational health services [38, 40], and absent occupational TB screening programs [27–29, 30, 40, 52, 54]. At times, TB IPC and occupational health policy implementation was viewed as a lower priority than clinical work [49].

#### Theme 4: Health worker perceptions of patient responses to TB IPC

Many health workers described patients as non-compliant with the TB IPC measures that they initiated [27, 33, 37, 40, 42, 48, 50, 51, 54–56]. Health workers framed this as a knowledge deficit [57] which they tried to correct through providing health education or pamphlets, but that patients did not adhere to nor understand [44]. A South African nurse said: ‘*Most of the [TB] patients are stubborn and their behaviour is uncooperative*. *… They have a bad attitude*, *don’t want to eat*, *carelessly cough around*, *refuse their tablets*. *They are a risk for everyone*. *Often I don’t trust them*’ [50]. Health workers distrusted TB patients because they felt patients might pretend to have TB [50] or use coughing as way to skip the queue [28]. Some health workers felt patients were deliberately trying to infect them ‘*through wicked strategies*’ like putting their sputum in the health worker’s food [43]. One counter-example was cited where patients reminded nursing staff to wear their protective equipment [27]. Tudor and collegues described similar narratives of ‘deviant patients’ not ‘complying’ and observed that this may be a way for health workers to shift the blame for TB transmission in healthcare settings from health workers to patients [51].

Yet health workers also considered stigma to be an important impediment to using TB IPC measures [21, 28, 30–32, 36, 39, 56, 57] (Table 2, **Key finding 7**) Specifically, they viewed patients to as feeling stigmatised by TB IPC measures, such as being singled out to wear a mask for source control [44]. Brouwer and colleagues reflected on this, noting that masks can have ‘*an alienating or depersonalizing effect and reduce the HCWs’ ability to provide compassionate care*’ [28]. Similarly, health workers struggled to decide between closing their consultation room door to respect the privacy of patients, or leaving an open door to improve ventilation [47]. Reducing the stigma associated with wearing a mask or a respirator was highlighted as an IPC priority [28], with one study suggested universal mask-wearing by health workers and patients as potential solution [36].

Instead of looking at ways to mitigate the stigma associated with wearing a mask, some health workers did not use PPE and avoided prolonged contact with patients. A physician from the Dominican Republic said: “*It is more cultural than anything*. *I put on a mask as if I am going to become infected [by a patient]*. *It looks ugly*, *so we do not do this*. *We do*, *however*, *keep our distance from patients and avoid speaking closely face-to-face*” [30]. Health workers avoided entering the rooms of patients with TB or spent the shortest possible time there [39]. They also avoided making patients wait during the consultation while they fetched PPE [29], deeming it acceptable to ‘*rather endanger [their] lives in order to save lives*’ [41]. Chapman and collegues offered an alternative to framing TB IPC to be in opposition to providing compassionate care. Instead, they believe health workers ‘*who fail to adhere to recommended M*. *tuberculosis infection control measures may not be upholding their ethical and moral responsibility to protect their own as well as their patients’ health*’ [30].

Dodor and colleagues drew a distinction between using IPC measures rationally (to prevent becoming infected) and using it irrationally (out of fear, when it is not needed, when is stigmatising and not effective in reducing transmission) [43]. From the overall dataset contributing to this theme, we noticed that this distinction is based on the underlying assumptions that health workers and researchers make about what constitutes ‘effective’ IPC measures. While effectiveness was not the focus of the review, there is evidence to suggest that patients are concerned about the spread of TB in health facilities, and that their views towards TB IPC measures might be more nuanced than health workers perceive [57].

#### Theme 5: Underlying uncertainties that influence how TB IPC tools are used

We indentified three important uncertainties that health workers described with regards to TB IPC implementation. Firstly, health workers described prioritising TB IPC implementation in clinical areas where patients known with TB or DR TB were seen, in particular, TB wards and DOTS centres [21, 29, 32–34, 36, 41, 42, 45, 47, 48, 50, 52–55] By contrast, housekeeping, administrative staff and community health workers were overlooked, as well as health workers providing undifferentiated patient care. (Table 2, **Key finding 8**) This targeting of TB IPC resources also demonstrated localisation of TB risk, with the contribution of undiagnosed people with TB to transmission being underappreciate both by health workers and researchers undertaking the studies. Kallon and collegues described what they observed in primary health care facilities in South Africa: ‘*These acts of locating risks in specific physical spaces within the facility or associating it with exposure to particular individuals emerged as an important shared*, *implicit knowledge in many facilities*. *It was not always clear whether HCWs really believed TB risk could so easily be located and managed*, *or they believed this inadequate approach was the only practical one available to them (or some mix of the two)’* [36]. They note that with an airborne illness, locating risks only to certain areas in a health care facility (the TB room) or certain patients (those with drug-resistant TB) is not an effective or logical use of TB IPC resources [36].

The second uncertainty that was prominent in our data relates to duration of infectiousness. Health workers described a wide variation in the duration over which that they perceived people with TB to be infectious [32, 43, 50] (Table 2, **Key finding 9**). This ranged from deeming patients less infectious immediately after starting treatment, to continuing infection control measures for the duration of TB treatment [50]. This has important implications for TB IPC resource allocation in facilities.

Thirdly, health workers described uncertainty about the modes of TB transmission.

They used a variety of IPC measures aimed at preventing the spread of TB, including contact, droplet and airborne precautions [23, 28, 34, 40, 42, 44, 46, 47, 49, 54, 56]. (Table 2, **Key finding 10**). While WHO guidelines describe TB as exclusively transmitted by the airborne route [10], some of the researchers and health workers focussed on droplet and contact precautions [45, 56] including ‘*showering and changing clothes so they did not carry the bacillus home*’ [35]. Health workers placed detailed focus on containing and disposing expectorated sputum—for example closing the sputum bottle to prevent it from being aerosolised as it dries [43], asking patients to bring a tin with sand to cough in [28], prefilling the sputum cup with water [49], putting a disinfectant in the sputum cup [46], or requiring separate bins for Tuberculosis waste [56].

We found other examples of TB IPC measures being implemented outside of what is recommended in international guidelines, this is described in Table 3.

**Table 3:** Contextualising and evaluating TB IPC implementation considerations identified through this qualitative evidence synthesis.

WHO TB IPC recommendation [10] and *implementation considerations included in the 2019 WHO TB IPC guideline*	Evaluation of implementation consideration identified through review. The headings and * indicate the interpretation of review authors.	Illustrative quotes
**Administrative controls**		
Triage people with TB signs and symptoms, or with TB disease ‘to fast-track TB diagnosis and facilitate further separation or other precautions.’ *‘Interventions within the three-level hierarchy of IPC should not be prioritized individually or implemented separately*, *but must be considered as an integrated package*.*’*	ASSUMPTION: Health workers assume patient triage requires a health worker to observe patient symptoms.	‘You just hear somebody coughing when you are here in the room, when you get out, you can’t even know who has been coughing. Maybe you ask and it may look embarrassing to a patient … So, it needs somebody who can sit there to just observe them’ [44].
	MISCONCEPTION: Because health workers believe drug-resistant TB to be caused by patients who ‘default’ treatment, they consider detection of treatment non-compliance an important component of triage for potential drug-resistant TB. *While treatment interuption may be a risk factor for drug resistant TB, the majority of patients aquire drug-resistant TB through transmission and will have no history of previous TB treatment—see for example the work of Shah [58].	‘We can suspect an MDR type of situation… We usually find that out ourselves. And that takes a bit of time when the person is sitting in a normal ward whatever the case is, and potentially has defaulted treatment’ [42].
Respiratory separation / isolation of people with presumed or demonstrated infectious TB *‘For the adequate implementation of isolation*, *it is important that health care authorities and those implementing the interventions consider the rights and freedoms of TB patients*, *balancing such individual liberties with the advancement of the common good*. *Facilities should meet minimal standards for implementation; staff should be trained; and the undesirable effects for those affected should be considered*.*’*	WORKAROUND: Health workers describe isolating immunocompromised patients instead of infectious patients when there is a shortage of isolation space.	‘Most of the participants from wards with TB routine admitted that periodically their isolation rooms were used for immuno-suppressed patients instead of those with infectious TB. “We keep the TB-patients in the front in order to protect the haematological patients in the back”‘ [50].
	MISCONCEPTION: Health workers interpret patient “isolation” to require fencing off the TB ward and restricting movement of TB patients.	‘There is a fence around the TB ward which restricts the movement of TB patients. Patients are only allowed to go out when it is necessary and then the appropriate precautionary measures are put in place’ [23].
Prompt initiation of effective TB treatment of people with TB disease is recommended to reduce M. tuberculosis transmission to health workers, persons attending health care facilities or other persons in settings with a high risk of transmission. *‘Treatment of patients needs to be guided by the use of drug-susceptibility testing*, *something that is important for field practitioners and implementers when putting these recommendations into practice*.*’*	UNCERTAINTY: Health workers describe being unsure how long after treatment initiation TB patients are not infectious and use very different time guidelines. *The duration of infectiousness of people with TB after starting TB treatment is also not clear in the WHO TB IPC guidelines [10].	‘Wrongly believing that the patient is immediately rendered non-infectious on commencement of treatment, some participants, a few from wards with TB-routine, immediately stopped applying the appropriate IPC measures (transmission- based precautions) “…once the first pills are swallowed”. Others did not trust the effect of treatment and continued transmission-based precautions for the entire period of patient admission’ [50].
Respiratory hygiene (including cough etiquette) in people with presumed or confirmed TB is recommended to reduce M. tuberculosis transmission *‘There is a need to provide health education to key stakeholders*, *effective health counselling for patients*, *use respiratory hygiene (including cough etiquette) as a standard practice for coughing patients and provide “how to” information on wearing of surgical masks*, *during sensitization and educational activities with both patients and health workers*.*’*	MISCONCEPTION: Health workers consider fomite transmission (through contaminated paper tissues) to be important to prevent TB transmission. *TB is broadly considered to spread exclusively through the airborne route.	‘We tell them handkerchief is better as they won’t dispose the handkerchief anyhow, unlike tissue paper which they will throw anywhere and the wind will blow the germ in any direction’ [54].
**Environmental controls**		
Upper-room germicidal ultraviolet (GUV) systems are recommended to reduce M. tuberculosis transmission *‘Because of cost considerations*, *the implementation of this intervention may not be feasible in all settings*. *Low- and middle-income countries that do not have the infrastructure or capacity to fully adopt this recommendation are advised to identify areas of higher risk of transmission*, *and prioritize the application of this intervention accordingly*.*’*	UNCERTAINTY: Health workers considered a poorly maintained GUV system to be risky, as it may result in staff thinking they are protected when they are not. They are therefore uncertain about the value of GUV systems as a preventative tool.	‘Sometimes it’s not so much of the technology but how it is applied. They (UV lights) were wrongly administered from an engineering point of view. Others at the wrong angle, not maintained, others the bulb not at the right UV emissions, wavelength’ [38].
Ventilation systems (including natural, mixed-mode, mechanical ventilation and recirculated air through HEPA filters) are recommended to reduce M. tuberculosis transmission. *‘The use of poorly designed or poorly maintained ventilation systems*, *leading to inadequate airflow*, *can result in health care- associated transmission of M*. *tuberculosis*.*’*	ASSUMPTION: Health workers described the only available ventilation options in low resource settings as opening windows, with its use limited by thermal discomfort. *Other options, such as upper-room GUV and HEPA filters are assumed to be inappropriate.	‘Some windows are bolted shut. Others are never opened because nobody [wants to] get cold in winter’ [25].
**Personal protective equipment**		
Particulate respirators, within the framework of a respiratory protection programme, are recommended to reduce M. tuberculosis transmission to health workers. *‘In line with international standards on occupational safety and health*, *it is imperative that national health care authorities make use of particulate respirators for health workers only when a respiratory protection programme can be put in place*. *… Effective implementation involves employee education and training activities on the proper use and maintenance (including repair and disposal) of particulate respirators*, *and periodic audits of practice*.*’*	MISCONCEPTION Some health workers believe wearing an N95 respirator on top of a surgical mask offers improved protection. *This may negatively influence the seal that the respirator forms around the wearer’s face.	‘The fear [of being infected with occupational TB] even induced the unsafe and inefficient method of IPC by wearing binary masks (respiratory and surgical masks) and hand gloves’ [34].
	MISCONCEPTION: Some health workers consider shorter periods of patient contact to require less or no respiratory protection when managing patients who may have infectious TB. *While the duration of exposure required for TB infection is uncertain, health workers are repeatedly exposed to TB at work and should always use precautions when they are managing someone with potential infectious TB.	‘When I go to see a patient in isolation room I don’t discuss; I just go and examine the patient. So when contact period is extremely short; triple layer [mask] is fine. But if you are going there and examining, spending more than 10–15 minutes than you cannot wear triple layer mask. It will become a compromise’ [46].
	MISCONCEPTION: Some health workers believe TB is more transmissible in the morning, thus requiring respiratory protection only at specific times. *While taking an early morning sputum specimen to diagnose TB may increase diagnostic yield, this does not translate to TB being less infectious at certain times of the day.	‘TB was concentrated in the morning air, but dissipated later in the day as patients moved in and out of the room, and therefore N95 masks should be worn in the morning, but not necessarily at other times’ [40].

## Discussion

This meta-synthesis has found that successful TB IPC implementation requires a multifaced approach that acknowledges complexity. Important findings, that we discuss in further depth in the sections that follow, include:

1. Health workers tasked with TB IPC implementation operate in a high stress environment where they feel exposed to occupational hazards. Whether they have received TB IPC training, how they view their personal risk of developing occupational TB and whether they feel empowered to influence their workplace safety all contribute to whether they are able to implement TB IPC.
2. TB IPC technologies pose difficulties of acceptability (for example that mask wearing may be stigmatising), and usability (for example discomfort associated with opening windows during cold weather). These are compounded by difficulties in procurement (for example of particulate filter respirators) and maintenance (for example of GUV systems).
3. There are key gaps both in TB IPC and occupational health policy content and local translation.
4. Health workers viewed patients as unsupportive of TB IPC measures, and described forgoing the use of some of the TB IPC tools to prevent stigmatising patients. However, this should be balanced with their ethical obligation to create a safe healthcare environment.
5. Health workers and TB IPC researchers have multiple uncertainties, assumptions and misconceptions relating to what constitutes TB IPC as intervention, and what is effective to curb TB transmission. Local TB implementation plans would help health workers navigate how to translate guidelines to their context.

We found that there is a complex interaction between TB IPC training, the risk perceptions of health workers of developing occupational TB, and workplace safety. This may explain why some health workers feel ambivalent or dismissive about TB IPC: where training is not accompanied by adequate resources or broader efforts to improve workplace safety, health workers may feel powerless with relation to influencing their working environment. This links with Nathavitharana’s argument, that TB IPC training should also motivate health workers to view themselves as ‘agents of change’ [59]. Factors related to health workers’ jobs—the high stress environment, health workers feeling unsafe or being a junior member of staff being delegated to work in the TB ward—all contribute to the “cognitive load” that influences TB IPC implementation. This often occurs within a broader context where organisational norms do not support the use of TB IPC. TB IPC implementation strategies that only target health worker knowledge of TB IPC will therefore unlikely be effective. Health workers need support as they try to balance TB IPC implementation with other competing clinical tasks, when negotiating TB IPC with colleagues, and through strategies that promote workplace safety more broadly.

Our data also emphasised the complexity involving TB IPC implementation, partly due to it multiple subcomponents. Yet there is also complexity inherent to the technology (for example the acceptability and usability of Germicidal UV systems) but also as part of broader processes within the health system, such as procurement and maintenance. Adapting work-flows related to infectious patients require collaboration with multiple organisational role players. Many of the research papers in our review reduced the complexity of implementing TB IPC to poor availability of TB IPC resources and infrastructure. While this is an important consideration, implementation guidelines should also consider other contributors to sub-optimal use of TB IPC tools. The NASSS-CAT framework could guide identifying complexities relating to TB IPC with prompts relating to TB as condition, the technologies used for TB IPC, its value proposition, how health workers view TB IPC measures, the organisation in which it is introduced and external context for innovation [60]. For example, when considering particulate filter respirators as technology, the framework prompts considering how the usability of particulate filter respirators could be improved through different respirators designs [61], strengthening supply chain management and whether using respirators disrupts organisational routines.

Our review also demonstrated important gaps in TB IPC and occupational health policy and implementation. This included contradictory guidelines, lack of prioritisation (for example implementation not being funded) and lack of local translation of national or international policy (for example addressing who is driving implementation and how it fits with existing workflows). With occupational health policy, health workers were unsure whether TB is an occupational disease and what an ideal occupational health service for TB should entail. Despite these uncertainties, health workers recommend linking TB IPC with occupational health services, a call consistent with statements by the International Labour Organisation and WHO [62, 63]. Yet this may position TB IPC implementation as a decision made by health workers about their own risk of developing TB. It may occur at the expense of practicing TB IPC as part of patient safety, as Colvin and colleagues noted [64]. In our evidence synthesis, health workers described patients as being ‘uncompliant’ and unsupportive of TB IPC. They did not view patients as important beneficiaries of TB IPC. In its extreme versions, the narratives of the ‘wicked’ and ‘unco-operative’ TB patient and the ‘heroic’ health worker forgoing PPE to provide care, seeks to create a moral distinction between health workers and patients which ultimately impedes TB IPC. Overall, these dilemmas need to be recognised and health workers need support in finding a balance between their concerns about TB IPC leading to stigma, the ethical responsibility to prevent the spread of TB in health facilities and prioritising the health of health workers.

We were struck by the large variation in TB IPC measures that were described, with health worker uncertainties, assumptions and misconceptions leading to less effective TB IPC being prioritised. This was most prominent in the targeting of TB IPC measures at patients known with TB, who are already on treatment and in many cases, no longer infectious [36]. While a TB diagnosis in a patient may be the first trigger health workers to consider IPC, WHO TB IPC guidance from as early as 1999 stated that people with TB symptoms who have not yet been diagnosed and not yet started treatment pose a higher risk of transmission than patients on treatment [65]. Although reiterated in the 2009 and 2019 WHO TB IPC guidance [10], based on our evidence synthesis findings this concept has had limited uptake in many high TB burden countries. A major shift in where TB IPC resources is directed is required–away from TB programmes and towards areas in health facilities in high TB burden settings where “undifferentiated” patients are seen. For example, health workers and administrative staff working in poorly ventilated waiting rooms and emergency care areas may be highly exposed to infectious TB and thus in need of IPC training and resources. We were alarmed that certain groups of health workers (for example housekeeping staff and community health workers) were not prioritised for TB IPC training and protective equipment. Furthermore, the dual applicability of airborne IPC measures to COVID-19 and TB, and potential transmission from asymptomatic patients for both disease warrants consideration of how airborne precautions could be better integrated [66, 67].

Our evidence synthesis described several other ways in which existing resources are being used suboptimally. One example is the emphasis health workers placed on sputum disposal, which is likely based on a theory prominent prior to the 1990s that TB transmission occured through sputum drying up and re-aerosolising [68]. It is now widely accepted that the exclusive mode of TB transmission is through the air, by aerosols being produced by someone infected with TB who coughs, speaks or breathes [68]. TB IPC implementation plans can play an important role is guiding health workers through these contested areas of TB IPC implementation. In Table 4 we suggest how the ten key findings of this review can be used as a starting point for developing context-specific TB IPC implementation plans. This can help direct local TB IPC resources to be most effective at preventing the spread of TB.

**Table 4:** Recommendations for translating the ten key findings of this qualitative evidence synthesis to TB IPC implementation plans.

**(1) Engage with health worker risk perceptions** Measuring and communicating TB disease and infection rates in health workers makes an important contribution to health workers’ perception of TB risk and the importance they attach to TB IPC. (*See CerQUAL key finding 1*, *high confidence)* When developing TB IPC training for health workers, incorporating stories of health workers who developed TB and incidence rates of occupational TB may influence whether health workers use TB IPC measures.
**(2) Link TB IPC with health worker safety** TB IPC implementation plans should also be linked with broader initiatives to improve health worker safety and working conditions. *(See CerQUAL key finding 2*, *moderate confidence)* Offering occupational health services for health workers to screen for and manage the works aspects of occupational TB can contribute to this.
**(3) Consider how TB IPC influences TB care** When health workers feel unsafe, they may use unnecessary or stigmatising TB IPC measures. *(See CerQUAL key finding 3*, *moderate confidence)* TB IPC implementation plans should explore local solutions to address stigma associated with TB IPC measures and how adverse impacts of TB IPC on care can be mitigated or negotiated.
**(4) Ensure reliable access to PPE** Health workers describe significant difficulty in reliably accessing particulate filter respirators, with some health worker groups being systematically excluded for higher grade PPE. *(See CerQUAL key finding 4*, *high confidence)* TB IPC implementation plans should include strategies for financing and procuring particulate filter respirator stock for all health workers working in areas of high risk to TB exposure.
**(5) Improve health facility ventilation and isolation infrastructure** Health workers described poor ventilation and isolation infrastructure in health facilities. *(See CerQUAL key finding 5*, *high confidence)* TB IPC implementation planning could start with health infrastructure directives that include minimum ventilation standards for new healthcare facilities, and a strategy to progressively upgrade existing facilities to ensure adequate ventilation.
**(6) Record and release occupational TB rates in health workers** Health workers expressed uncertainty about whether TB is an occupational disease in health workers. *(See CerQUAL key finding 6*, *low confidence)* Depending on context (for example countries with a high TB burden where TB is legally defined as an occupational disease), a national occupational TB register could contribute to creating awareness of TB as occupational disease which may facilitate tracking occupational TB as indicator of poor TB IPC implementation.
**(7) Stigma caused by TB IPC measures should be mitigated** Stigma is described as one of the main barriers to using TB IPC measures more widely in health facilities, especially the use of masks as source control. *(See CerQUAL key finding 7*, *high confidence)* Both on national and local implementation level, mitigating this stigma should be discussed. For example, by reviewing contextual factors (local TB epidemiology, rates of asymptomatic TB transmission and mask availability) policymakers could consider universal mask wearing in health facilities for TB which can be less stigmatising than targeted mask wearing.
**(8) Shift TB IPC resources from targeting patients known with TB, to high-risk areas** Health workers commonly focus TB IPC measures on patients who are known with TB, already on treatment. *(See CerQUAL key finding 8*, *high confidence)* To address this common implementation gap, TB IPC implementation plans should focus on high-risk areas where undifferentiated patients are seen, prior to diagnosis and treatment initiation for TB. Health worker TB IPC training and resources should be provided to all frontline workers in high TB burden settings, not only those providing care for patients with TB or drug-resistant TB. Special care should be taken to include staff working in housekeeping, administration, students and community health workers.
**(9) Specify duration of infectiousness for TB** There is wide variation in the duration that health workers perceive patients with TB to be infectious. *(See CerQUAL key finding 9*, *low confidence)* This may also contribute to the stigmatisation of patients with DR-TB. TB IPC implementation plans should include a best estimate of the duration of infectiousness of people with TB, and guidance on how to relate diagnostic tests to infectious risk (for example repeat smear microscopy).
**(10) Explicitly recommend against ineffective TB IPC measures** Health workers use a variety of measures for TB IPC, some of which are ineffective (for example contact or droplet precautions). *(See CerQUAL key finding 10*, *moderate confidence)* TB IPC implementation plans should include negative recommendations against unnecessary or ineffective measures (for example elaborate sputum disposal) with the goal of using limited TB IPC resources effectively.

Our review findings are distinct from those of two previous reviews of TB IPC implementation as we focussed only on qualitative research, strove to describe complexity and aimed to inform implementation plans. Our inductive approach is different from the two more deductive approaches of the reviews by Tan and colleagues—who used a macro, meso, micro framework [9]—and that of Zwama and colleagues that used a health systems framework to code their data [14]. Our key findings elaborate on the exisiting implementation recommendations in the 2019 WHO TB IPC guidelines [10] that we described in Table 4.

For our understanding of the subject to progress, we belive that TB IPC qualitative research should shift from merely documenting poor TB IPC implementation in different settings, often through lists of barriers and facilitators. Instead, it could explore ways in which common obstacles to IPC are navigated, why some facilities are successful in implementing TB IPC, or how staff respond to interventions to improve TB IPC. There is a need to examine the complex impacts of TB IPC as an intervention. As Brennan describes, ‘*An intervention aimed at a particular function will reverberate across the whole system*. *Innovation is always accompanied by unpredictability and unintended consequences*, *by positive and negative feedback loops*. … *It is the task of research to trace and explain such emergent effects*’[69]. For example, across different settings, health workers commonly described particulate filter respirators as difficult to wear. Future research should study whether there are differences in acceptability between respirator design, whether acceptability improves over time, under which conditions health workers would be willing to use respirators as part of universal precautions, and how respirators influence their ability to provide care. Finally, few of the qualitative studies to date have used theory or conceptual frameworks to interpret their findings. Linking qualitative findings with existing social science theory would add additional depth to descriptions and interpretations.

A strength of this review is that we brought an interdisciplinary perspective to the topic and focussed on finding pragmatic strategies to support health workers as they try to implement TB IPC. A limitation of this review is that it draws on research that was conducted in low- and middle-income countries, mostly with a high TB burden. The findings may therefore not be relevant to low TB burden, high income settings. We did not include grey literature or abstracts of articles that were not available in English in our review. It is possible that we missed anthropological accounts of health worker TB IPC measures that did not include ‘infection prevention and control’ in the title. Despite this, we believe that drawing on 37 qualitative research papers from different settings strengthened the transferability of the findings of our review.

## Conclusion

A common refrain from the qualitative studies reviewed here is that TB IPC is poorly implemented in most high TB burden settings. In order to progress towards solutions that support health workers as they attempt to address this, we need a better understanding of TB IPC as a complex intervention that depends on multiple subcomponents. We need to notice which underlying principles are being contested or misinterpreted (for example where should the bulk of TB IPC efforts be directed). The healthcare settings in which TB IPC is most needed are often poorly resourced, with health workers working under high cognitive load. Health workers need support as they navigate such working environments to try to implement TB IPC as an intervention. Finding ways to adapt TB IPC implementation plans to specific settings can play an important role in providing the support health workers need.

## Supporting information

S1 Appendix Searches run. (DOCX)

S2 Appendix CERQual evidence profile. (DOC)

S1 Table Summary of included studies. (DOCX)

S2 Table Critical appraisals skills programme scores of included studies. (DOCX)

S3 Table ENTREQ checklist. (DOCX)
