# Supplementary material for: Health worker experiences of implementing TB infection prevention and control: A qualitative evidence synthesis to inform implementation recommendations
Source: PLOS Glob Public Health. 2022 Jul 7;2(7):e0000292. doi: 10.1371/journal.pgph.0000292 (PMC10021216; doi:10.1371/journal.pgph.0000292)
Supplement: S1 Appendix — (DOCX) [file pgph.0000292.s001.docx]

**S2 Appendix: Searches run**

*These searches were run on 23 November 2021*

Medline (Ovid MEDLINE® Epub Ahead of Print, In-Process & Other Non-Indexed Citations, Ovid MEDLINE® Daily and Ovid MEDLINE®) [1946 to present]

| **# ▲** | **Searches** |  |
| --- | --- | --- |
| 1 | exp Health Personnel/ | 564582 |
| 2 | (health* adj2 (worker? or staff* or personnel)).mp. | 248381 |
| 3 | (doctor? or physician?).mp. | 692716 |
| 4 | nurse?.mp. | 367373 |
| 5 | (community adj2 (worker? or staff* or personnel)).mp. | 11531 |
| 6 | Occupational Exposure/ | 58115 |
| 7 | ((occupation* or work*) adj5 (risk? or hazard? or exposure?)).mp. | 113571 |
| 8 | 1 or 2 or 3 or 4 or 5 or 6 or 7 | 1446606 |
| 9 | exp Tuberculosis/pc, tm [Prevention & Control, Transmission] | 24105 |
| 10 | exp Tuberculosis/ | 199041 |
| 11 | (tuberculosis or tb or mdrtb).mp. | 283791 |
| 12 | 10 or 11 | 285784 |
| 13 | infection control/ or patient isolation/ | 31555 |
| 14 | Cross Infection/ | 59401 |
| 15 | disease transmission, infectious/ or infectious disease transmission, patient-to-professional/ or infectious disease transmission, professional-to-patient/ | 17525 |
| 16 | Risk Factors/ | 898001 |
| 17 | Risk Reduction Behavior/ | 13858 |
| 18 | ((infection or tuberculosis or tb or mdrtb) adj3 (prevent* or control*)).mp. | 106616 |
| 19 | (risk? adj3 (reduc* or control*)).mp. | 198587 |
| 20 | isolat*.mp. | 2144815 |
| 21 | 13 or 14 or 15 or 16 or 17 or 18 or 19 or 20 | 3257367 |
| 22 | 12 and 21 | 60246 |
| 23 | 9 or 22 | 71888 |
| 24 | (implement* or impact* or capacity).mp. | 2365583 |
| 25 | (barrier? or challeng* or obstacle* or concern* or worry or worries).mp. | 1959629 |
| 26 | (facilitat* or empower* or influenc* or enabl* or motivat*).mp. | 2790319 |
| 27 | (experience? or perspective? or perception? or attitude? or view*).mp. | 2612141 |
| 28 | 24 or 25 or 26 or 27 | 7791261 |
| 29 | grounded theory/ or exp qualitative research/ | 71066 |
| 30 | focus groups/ or interviews as topic/ | 95143 |
| 31 | (qualitative or interview* or focus group* or mixed method? or phenomenolog* or ethnograph* or grounded theory or participant observation).mp. or observation.ti. | 700005 |
| 32 | ((openended or open-ended or unstructured or semi-structured or semistructured) adj3 (question* or survey?)).mp. | 17861 |
| 33 | 29 or 30 or 31 or 32 | 707306 |
| 34 | 8 and 23 and 28 and 33 | 525 |

**Embase** (OvidSP) [1974-present]

| **# ▲** | **Searches** | **Results** |
| --- | --- | --- |
| 1 | exp health care personnel/ | 1722242 |
| 2 | (health* adj2 (worker? or staff* or personnel)).mp. | 324918 |
| 3 | (doctor? or physician?).mp. | 952967 |
| 4 | nurse?.mp. | 431950 |
| 5 | (community adj2 (worker? or staff* or personnel)).mp. | 10223 |
| 6 | Occupational Exposure/ | 85347 |
| 7 | ((occupation* or work*) adj5 (risk? or hazard? or exposure?)).mp. | 158319 |
| 8 | 1 or 2 or 3 or 4 or 5 or 6 or 7 | 2511128 |
| 9 | tuberculosis control/ | 6792 |
| 10 | exp Tuberculosis/ | 203445 |
| 11 | (tuberculosis or tb or mdrtb).mp. | 272731 |
| 12 | 10 or 11 | 282435 |
| 13 | infection control/ or patient isolation/ | 94779 |
| 14 | Cross Infection/ | 20041 |
| 15 | disease transmission/ or vertical transmission/ | 120533 |
| 16 | Risk Factor/ | 1164567 |
| 17 | Risk Reduction/ | 114516 |
| 18 | ((infection or tuberculosis or tb or mdrtb) adj3 (prevent* or control*)).mp. | 226313 |
| 19 | (risk? adj3 (reduc* or control*)).mp. | 337061 |
| 20 | isolat*.mp. | 2027289 |
| 21 | 13 or 14 or 15 or 16 or 17 or 18 or 19 or 20 | 3675991 |
| 22 | 12 and 21 | 69029 |
| 23 | 9 or 22 | 69029 |
| 24 | (implement* or impact* or capacity).mp. | 3213520 |
| 25 | (barrier? or challeng* or obstacle* or concern* or worry or worries).mp. | 2508206 |
| 26 | (facilitat* or empower* or influenc* or enabl* or motivat*).mp. | 3368660 |
| 27 | (experience? or perspective? or perception? or attitude? or view*).mp. | 3323063 |
| 28 | 24 or 25 or 26 or 27 | 9859054 |
| 29 | grounded theory/ or naturalistic inquiry/ or qualitative research/ | 99695 |
| 30 | exp *interview/ | 16207 |
| 31 | (qualitative or interview* or focus group* or mixed method? or phenomenolog* or ethnograph* or grounded theory or participant observation).mp. or observation.ti. | 899025 |
| 32 | ((openended or open-ended or unstructured or semi-structured or semistructured) adj3 (question* or survey?)).mp. | 24885 |
| 33 | 29 or 30 or 31 or 32 | 914723 |
| 34 | 8 and 23 and 28 and 33 | 683 |

**Global Health** (OvidSP) [1973-2021 Week 46]

| **# ▲** | **Searches** |  |  |
| --- | --- | --- | --- |
| 1 | exp health care workers/ | | 83387 |
| 2 | (health* adj2 (worker? or staff* or personnel)).mp. | | 58550 |
| 3 | (doctor? or physician?).mp. | | 64439 |
| 4 | nurse?.mp. | | 25416 |
| 5 | (community adj2 (worker? or staff* or personnel)).mp. | | 4750 |
| 6 | ((occupation* or work*) adj5 (risk? or hazard? or exposure?)).mp. | | 45062 |
| 7 | 1 or 2 or 3 or 4 or 5 or 6 | | 178241 |
| 8 | exp mycobacterium tuberculosis/ | | 60625 |
| 9 | (tuberculosis or tb or mdrtb).mp. | | 69057 |
| 10 | 8 or 9 | | 69057 |
| 11 | infection control/ or disease control/ | | 58292 |
| 12 | Cross Infection/ | | 485 |
| 13 | disease transmission/ or vertical transmission/ | | 69907 |
| 14 | Risk Factors/ | | 271564 |
| 15 | Risk Reduction/ | | 17340 |
| 16 | ((infection or tuberculosis or tb or mdrtb) adj3 (prevent* or control*)).mp. | | 45973 |
| 17 | (risk? adj3 (reduc* or control*)).mp. | | 65513 |
| 18 | isolat*.mp. | | 372021 |
| 19 | 11 or 12 or 13 or 14 or 15 or 16 or 17 or 18 | | 789320 |
| 20 | 10 and 19 | | 26084 |
| 21 | (implement* or impact* or capacity).mp. | | 472056 |
| 22 | (barrier? or challeng* or obstacle* or concern* or worry or worries).mp. | | 327838 |
| 23 | (facilitat* or empower* or influenc* or enabl* or motivat*).mp. | | 386009 |
| 24 | (experience? or perspective? or perception? or attitude? or view*).mp. | | 324350 |
| 25 | 21 or 22 or 23 or 24 | | 1158966 |
| 26 | interviews/ | | 2908 |
| 27 | (qualitative or interview* or focus group* or mixed method? or phenomenolog* or ethnograph* or grounded theory or participant observation).mp. or observation.ti. | | 160056 |
| 28 | ((openended or open-ended or unstructured or semi-structured or semistructured) adj3 (question* or survey?)).mp. | | 6155 |
| 29 | 26 or 27 or 28 | | 162723 |
| 30 | 7 and 20 and 25 and 29 | | 417 |

**CINAHL** (EBSCOHost) [1982-present]

| **#** | **Query** | **Results** |
| --- | --- | --- |
| S22 | S5 AND S14 AND S15 AND S21 | 240 |
| S21 | S16 OR S17 OR S18 OR S19 OR S20 | 476,471 |
| S20 | TI ( qualitative or interview* or "focus group*" or "mixed method*" or phenomenolog* or ethnograph* or "grounded theory" or "participant observation" ) OR AB ( qualitative or interview* or "focus group*" or "mixed method*" or phenomenolog* or ethnograph* or "grounded theory" or "participant observation" ) OR TI observation OR TI ( ((openended or open-ended or unstructured or semi-structured or semistructured) N3 (question* or survey*)) ) OR AB ( ((openended or open-ended or unstructured or semi-structured or semistructured) N3 (question* or survey*)) ) | 352,596 |
| S19 | (MH "Narratives") | 19,181 |
| S18 | (MH "Focus Groups") | 47,051 |
| S17 | (MH "Interviews+") | 234,827 |
| S16 | (MH "Qualitative Studies+") | 163,124 |
| S15 | TI ( implement* or impact* or capacity ) OR AB ( implement* or impact* or capacity ) OR TI ( barrier* or challeng* or obstacle* or concern* or worry or worries ) OR AB ( barrier* or challeng* or obstacle* or concern* or worry or worries ) OR TI ( facilitat* or empower* or influenc* or enabl* or motivat* ) OR AB ( facilitat* or empower* or influenc* or enabl* or motivat* ) OR TI ( experience* or perspective* or perception* or attitude* or view* ) OR AB ( experience* or perspective* or perception* or attitude* or view* ) | 1,897,768 |
| S14 | S6 OR S13 | 10,453 |
| S13 | S9 AND S12 | 7,729 |
| S12 | S10 OR S11 | 426,679 |
| S11 | TI ( ((infection or tuberculosis or tb or mdrtb) N3 (prevent* or control*)) ) OR AB ( ((infection or tuberculosis or tb or mdrtb) N3 (prevent* or control*)) ) OR TI ( (risk* N3 (reduc* or control*)) ) OR AB ( (risk* N3 (reduc* or control*)) ) OR TI isolat* OR AB isolat* | 213,012 |
| S10 | (MH "Infection Control") OR (MH "Patient Isolation") OR (MH "Cross Infection") OR (MH "Disease Transmission") OR (MH "Disease Transmission, Patient-to-Professional") OR (MH "Disease Transmission, Professional-to-Patient") OR (MH "Risk Factors") | 244,730 |
| S9 | S7 OR S8 | 31,636 |
| S8 | TI ( tuberculosis or tb or mdrtb ) OR AB ( tuberculosis or tb or mdrtb ) | 26,613 |
| S7 | (MH "Tuberculosis+") | 23,647 |
| S6 | (MH "Tuberculosis+/PC/TM") | 5,252 |
| S5 | S1 OR S2 OR S3 OR S4 | 1,021,255 |
| S4 | TI ( ((occupation* or work*) N5 (risk* or hazard* or exposure*)) ) OR AB ( ((occupation* or work*) N5 (risk* or hazard* or exposure*)) ) | 26,629 |
| S3 | (MH "Occupational Exposure") | 20,158 |
| S2 | TI ( (health* N2 (worker* or staff* or personnel)) ) OR AB ( (health* N2 (worker* or staff* or personnel)) ) OR TI ( doctor* or physician* ) OR AB ( doctor* or physician* ) OR TI ( nurse or nurses ) OR AB ( nurse or nurses ) OR TI (community N2 (worker* or staff* or personnel)) OR AB (community N2 (worker* or staff* or personnel)) | 582,164 |
| S1 | (MH "Health Personnel+") | 604,558 |

**African Index Medicus** (via <https://www.globalindexmedicus.net/> )

(tw:(TB OR mdrtb OR tuberculosis)) AND (tw:(control OR "cross infection" OR transmission OR risk*)) AND (tw:(qualitative OR interviews OR "focus groups" OR "grounded theory" OR ethnography OR ethnographic OR phenomenology OR observation OR survey OR questionnaire OR questionnaires))

**Science Citation Index, Social Science Citation Index and Conference Proceedings Citation Index – Science** (Web of Science Core Collection)[1900-present]

| **No:** | **Hits:** | **Search terms:** |
| --- | --- | --- |
| # 6 | 359 | #5 AND #4 AND #3 AND #2 AND #1 |
| # 5 | 1,578,180 | TS=( qualitative or interview* or "focus group*" or "mixed method*" or phenomenolog* or ethnograph* or "grounded theory" or "participant observation" ) OR TI=observation OR TS=((openended or open-ended or unstructured or semi-structured or semistructured) N3 (question* or survey*)) |
| # 4 | 17,101,490 | TS= ( implement* or impact* or capacity ) OR TS= ( barrier* or challeng* or obstacle* or concern* or worry or worries ) OR TS=( facilitat* or empower* or influenc* or enabl* or motivat* ) OR TS=( experience* or perspective* or perception* or attitude* or view* ) |
| # 3 | 2,118,292 | TS=((infection or tuberculosis or tb or mdrtb) NEAR/3 (prevent* or control*)) OR TS=(risk* NEAR/3 (reduc* or control*)) OR TS=isolat* |
| # 2 | 229,270 | TS=( tuberculosis or tb or mdrtb ) |
| # 1 | 953,407 | TS=(health* NEAR/2 (worker* or staff* or personnel)) OR TS=( doctor* or physician* ) OR TS=( nurse or nurses ) OR TS=(community NEAR/2 (worker* or staff* or personnel)) OR TS=((occupation* or work*) NEAR/5 (risk* or hazard* or exposure*)) |
