# Supplementary material for: Health worker experiences of implementing TB infection prevention and control: A qualitative evidence synthesis to inform implementation recommendations
Source: PLOS Glob Public Health. 2022 Jul 7;2(7):e0000292. doi: 10.1371/journal.pgph.0000292 (PMC10021216; doi:10.1371/journal.pgph.0000292)
Supplement: S2 Appendix — (DOC) [file pgph.0000292.s002.doc]

| S4 Appendix: CERQual Evidence Profile | | | | | | | |
| --- | --- | --- | --- | --- | --- | --- | --- |
| Summary of review key findings | Studies contributing to the review finding | Methodological limitations | Coherence | Adequacy | Relevance | CERQual assessment of confidence in the evidence | Explanation of CERQual assessment |
| (1) Health workers describe their perception of their own TB risk as contributing to the importance they attach to TB IPC (e.g., hearing about other health workers who developed occupational TB) | Padayatchi *et al.*, 2010; Woith, Volchenkov and Larson, 2012; Adeleke, 2012; Arjun, Matlakala and Mavundla, 2013; Cowan *et al.*, 2013; Brouwer *et al.*, 2014; Daftary and Padayatchi, 2016; Chapman *et al.*, 2017b, 2017a; Zinatsa *et al.*, 2018; Probandari *et al.*, 2019; Kallon *et al.*, 2021 | Minor methodological limitations overall. Two of the 12 studies contributing to this finding had major methodological limitations (small sample size and unclear descriptions of methods) and one minor methodological limitations (limited discussion of findings). | No concerns about coherence. | No or very minor concerns about adequacy. | Moderate concern about relevance. Geographic spread includes six countries from Africa, the Americas, Europe and Asia. | High confidence | Data from 12 studies, three with methodological limitations. Studies form six different countries with wide geographic spread. |
| (2) Health workers describe feeling unvalued and unsafe at work, and that they have limited power over their working conditions. The risk of being exposed to TB is part of this insecurity. | Padayatchi *et al.*, 2010; Zelnick *et al.*, 2013; Bieh, Weigel and Smith, 2017; Chapman *et al.*, 2017b, 2017a; Van der Westhuizen and Dramowski, 2017; Zinatsa *et al.*, 2018; Marais, Kallon and Dudley, 2019; Matakanye, Ramathuba and Tugli, 2019; Probandari *et al.*, 2019; Adu *et al.*, 2020 | Minor methodological limitations. Two of the 11 studies had minor methodological limitations (unclear descriptions of methods). | No concerns about coherence. | No or very minor concerns about adequacy. | Major concern about relevance to countries not represented and limited geographic spread. Four countries represented, from Africa, the Americas and Asia. | Moderate confidence | Data from 11 studies, two with minor methodological limitations. Studies from four different countries, thus limited geographic spread. |
| (3) Health workers may stigmatise patients because of their fear of being exposed to TB and compromise clinical care by avoiding contact, shortening consultations, and restricting patient movement, e.g. through fencing wards and locking patient rooms. | Dodor and Kelly, 2010; Buregyeya *et al.*, 2013; Cowan *et al.*, 2013; Daftary and Padayatchi, 2016; Tamir, Wasie and Azage, 2016; Khaund, Sudhakar and Vaz, 2018; Kuyinu *et al.*, 2019; Matakanye, Ramathuba and Tugli, 2019; Probandari *et al.*, 2019; Fadare *et al.*, 2020 | Major methodological limitations. Of the ten studies, two had major methodological limitations (unclear description of methods) and four had moderate methodological limitations (one had an insufficient sample, one unclear findings section and one data analysis methods not described). | No concerns about coherence. | No or minor concerns about adequacy. | Moderate concern about relevance to countries not represented in the sample. Six countries represented, the majority from Africa, and two from Asia. | Moderate confidence | Data from 10 studies, six with methodological limitations. Studies predominantly from countries in Africa, limiting relevance. |
| (4) Health workers describe unavailability of particulate filter respirators as the main barrier to using respiratory protective equipment. Examples were given of doctors having preferential access over nurses or housekeeping staff when stock was limited. | Padayatchi *et al.*, 2010; Sissolak, Marais and Mehtar, 2011; Woith, Volchenkov and Larson, 2012; Arjun, Matlakala and Mavundla, 2013; Tudor *et al.*, 2013; Zelnick *et al.*, 2013; Cowan *et al.*, 2013; Brouwer *et al.*, 2014; Tamir, Wasie and Azage, 2016; Daftary and Padayatchi, 2016; Akshaya *et al.*, 2017; Chapman *et al.*, 2017a, 2017b; Marme, 2018; Khaund, Sudhakar and Vaz, 2018; Matakanye, Ramathuba and Tugli, 2019; Kuyinu *et al.*, 2019; Fadare *et al.*, 2020; Nazneen *et al.*, 2021 | Of the 19 studies contributing to this finding, four had major methodological limitations (two unclear methods, two insufficient sample size) and two moderate methodological limitations (limited sample size and insufficient explanation of data analysis). | No concerns about coherence. | No concerns about adequacy. | Minor concerns about relevance. Nine countries represented, from the Americas, Africa, Europe and Asia | High confidence | Data from 19 studies, six with methodological limitations. Studies from nine different countries. |
| (5) Health workers report working in facilities where the infrastructure was not designed for airborne infection control measures, with waiting areas, consultation rooms and wards lacking natural or mechanical ventilation and having an insufficient number of isolation rooms. | Sissolak, Marais and Mehtar, 2011; Arjun, Matlakala and Mavundla, 2013; Tudor *et al.*, 2013; Zelnick *et al.*, 2013; Buregyeya *et al.*, 2013; Cowan *et al.*, 2013; Brouwer *et al.*, 2014; Kuyinu *et al.*, 2016, 2019; Tamir, Wasie and Azage, 2016; Akshaya *et al.*, 2017; Maroldi *et al.*, 2017; Chapman *et al.*, 2017b, 2017a, 2018; Marme, 2018; Zinatsa *et al.*, 2018; Khaund, Sudhakar and Vaz, 2018; Marais, Kallon and Dudley, 2019; Matakanye, Ramathuba and Tugli, 2019; Adu *et al.*, 2020; Mwenya and Stapley, 2020; Nazneen *et al.*, 2021 | Of the 23 studies contributing to this finding, five had major methodological limitations (two insufficient sample size, three with unclear methods) and three minor methodological limitations (two with unclear data analysis and one with limited data) | No concerns about coherence. | No concerns about adequacy. | Minor concern about relevance. Twelve countries from Africa, Asia and the Americas contributed to this finding. | High confidence | Data from 23 studies, eight with methodological limitations. Studies from twelve different countries. |
| (6) Health workers express uncertainty about whether TB is an occupational illness and whether they needed to prove they were infected at work. This leads to underreporting of occupational TB. | Adeleke, 2012; Zelnick *et al.*, 2013; Zinatsa *et al.*, 2018; Adu *et al.*, 2020 | Moderate methodological limitations. Of the four studies included, one had major methodological limitations with unclear methods. | No concerns about coherence. | Major concerns about adequacy, only four studies contributing to this finding. | Major concerns about relevance as only one country (South Africa) represented. | Low confidence | Data from four studies, one with methodological limitations, all from South Africa. |
| (7) Health workers perceive patients as feeling stigmatised by TB IPC measures, such as being asked to wear a mask for source control. In general, health workers consider stigma an important impediment to implementing TB IPC measures. | Buregyeya *et al.*, 2012; Brouwer *et al.*, 2014; Daftary and Padayatchi, 2016; Bieh, Weigel and Smith, 2017; Chapman *et al.*, 2017a; Marme, 2018; Zinatsa *et al.*, 2018; Mwenya and Stapley, 2020; Kallon *et al.*, 2021 | Minor methodological limitations. One of the nine studies have minor methodological limitations (unclear methods). | No concerns about coherence | Minor concerns about adequacy, nine studies contributing to this finding. | Minor concerns about relevance, seven countries represented, from Africa, the Americas, Asia and Oceania. | High confidence | Data from nine studies, one study with minor methodological limitations and from seven different countries. |
| (8) Health workers describe the targeting of TB IPC resources towards clinical areas where patients with known TB or drug resistant TB are seen, including TB wards and DOTS centres. Health workers directly caring for such patients have priority access to personal protective equipment. Housekeeping, administrative staff and community health workers are frequently overlooked, as are health workers providing other types of patient care, despite the pervasiveness of TB risk. | Sissolak, Marais and Mehtar, 2011; Cowan *et al.*, 2013; Tamir, Wasie and Azage, 2016; Kuyinu *et al.*, 2016, 2019; Chapman *et al.*, 2017b, 2018; Maroldi *et al.*, 2017; Zinatsa *et al.*, 2018; Marme, 2018; Matakanye, Ramathuba and Tugli, 2019; Probandari *et al.*, 2019; Marais, Kallon and Dudley, 2019; Mwenya and Stapley, 2020; Fadare *et al.*, 2020; Nazneen *et al.*, 2021; Kallon *et al.*, 2021 | Minor methodological limitations overall. Of the seventeen studies contributing to this finding, two studies had minor methodological limitations (unclear sampling and small sample size) and three major methodological limitations (one with brief interviews and two with unclear methods of analysis). | No concerns about coherence. | No or only minor concerns about adequacy. | Minor concerns about relevance to countries with significant TB burden. Geographic spread: ten countries from Africa, the Americas and Asia. | High confidence | Data from seventeen studies, five with methodological limitations. Studies from ten different countries. |
| (9) There is a wide variation in the duration that different health workers perceive patients with TB to be infectious. | Dodor and Kelly, 2010; Sissolak, Marais and Mehtar, 2011; Zinatsa *et al.*, 2018 | Minor methodological limitations. One of the three studies had minor methodological limitations (unclear methods). | No concerns about coherence. | Major concerns about adequacy, only three studies contributing to this finding. | Major concern about relevance - studies from two countries both in Africa. | Low confidence | Data from three studies, minor methodological limitations, but only from two countries. |
| (10) Health workers describe using a variety of IPC measures aimed at preventing the spread of TB, including contact, droplet and airborne precautions. | Zelnick *et al.*, 2013; Buregyeya *et al.*, 2013; Brouwer *et al.*, 2014; Tshitangano, 2014; Kuyinu *et al.*, 2016, 2019; Akshaya *et al.*, 2017; Khaund, Sudhakar and Vaz, 2018; Marais, Kallon and Dudley, 2019; Probandari *et al.*, 2019; Mwenya and Stapley, 2020 | Moderate methodological limitations. Of the eleven studies included, four had major methodological limitations (methods not described, findings not based on sufficient data) and two minor methodological limitations (unclear findings section, unclear data analysis) | Minor concerns about coherence. Examples of assumptions and misconception derived from different settings. | No or only minor concerns about adequacy, ten studies contributing to this finding. | Minor concern about relevance, six countries represented, from Africa and Asia. | Moderate confidence | Data from eleven studies, six with methodological limitations from six countries. |
