# Supplementary material for: Health worker experiences of implementing TB infection prevention and control: A qualitative evidence synthesis to inform implementation recommendations
Source: PLOS Glob Public Health. 2022 Jul 7;2(7):e0000292. doi: 10.1371/journal.pgph.0000292 (PMC10021216; doi:10.1371/journal.pgph.0000292)
Supplement: S1 Table — (DOCX) [file pgph.0000292.s003.docx]

| **S2 Table: Summary of included studies** | | | | | | | |
| --- | --- | --- | --- | --- | --- | --- | --- |
| **Lead author**  **Country where study was based**  **(Year)** | **Title of Publication** | **Aims** | **Method of qualitative data collection and method of conducting qualitative analysis** | **Descriptive or explanatory theory or framework** | **Number of participants and facility type** | **Extract of key findings** | **CASP score**  **(out of 10)** |
| Adeleke  Khayelitsha, Western Cape, South Africa  (2012) | Barriers to implementation of Tuberculosis infection control amongst South African health care workers. | ﻿To provide an overview of health system factors that influence health worker performance in implementing TB infection control (TBIC) in primary care clinics at district level. | FGD and semi-structured interviews  No method of analysis described | None described | 3 key informant interviews, 7 semi-structured interviews and 3 focus group discussions at two primary health care clinics | ‘Bureaucratic delay in compensating HCWs (with active TB disease) is a major barrier to building trust in the health system. Such mistrust is an obstacle to sustained TBIC implementation among HCWs. Professional and lay HCWs are not implementing TBIC according to facility-specific policy. The last line of defence, PPE, was mostly prioritised by HCWs instead of administrative and environmental measures.’ | 3 |
| Adu  Gauteng, Western Cape and KwaZulu-Natal provinces of South Africa  (2020) | Perceived Health System Barriers to Tuberculosis Control Among Health Workers in South Africa | To elicit the percep­tions of informed persons of the health system barriers to achieving protection against occupational TB in health workers. | Semi-structured interviews  Unclear method of analysis, but likely framework approach. | World Health Organisation health system building blocks framework | 18 key informants ‘whose current position entailed the care, prevention, control and/or management of TB or were indirectly involved in TB prevention or policy’ | ‘The following health system barriers were identified by informants: leadership and governance were “top-down” and fragmented; lack of funding was a major barrier; there were insufficient numbers of staff trained in infection prevention and control and occupational health; occupational health services were not comprehensively available and the ability to sustain protective technologies was questioned. A cross-cutting barrier was lack of priority afforded to workforce occupational health associated with lack of accurate information on cases of TB among health workers.’ | 9 |
| Akshaya  Karnataka,  India  (2017) | “Who has to do it at the end of the day?  Programme officials or hospital authorities?”  Airborne infection control at drug resistant  tuberculosis (DR-TB) centres of Karnataka,  India: a mixed-methods study | To assess the compliance  of DR-TB centres to National AIC guidelines  (2010) and explore the provider perspectives related to  the barriers encountered for implementation in the state  of Karnataka, India (2016-17). | Key informant  Interviews using interview guides.  Content analysis was used to analyse interviews. | Developed inductive explanatory framework based on findings. | 20 health care providers participated including  administrators (n = 4), medical officers or nodal  officers (n = 4), staff nurses (n = 6) and housekeeping  employees (n = 6). Participants were based at six DR TB treatment centres. | ‘The reasons for unsatisfactory compliance to AIC guidelines were poor coordination between programme and hospital authorities leading to lack of ownership; ineffective or non-existent infection control committees; vacant posts of medical officers; and attitudes of health care delivery staff.’ | 4 |
| Arjun  KwaZulu Natal, South Africa  (2013) | Enrolled nurses' experiences of caring for multi drug resistant tuberculosis patients in the KwaZulu Natal province of South Africa | The purpose of this study was to explore and describe the experiences of enrolled nurses while caring for patients infected with MDR TB in a TB hospital in the KZN Province of South Africa. | Unstructured in-depth individual interviews.  Colaizzi’s seven steps used to analyse interviews. | Not used. | Five enrolled nurses at a MDR TB hospital. | ‘The enrolled nurses experienced fear of contracting MDR TB. The lack of equipment and supplies impacted negatively on providing quality nursing care. Although they sometimes received support from management, it was insufficient, but they received valuable support from their colleagues. The enrolled nurses also expressed their needs for in-service education and occupational compensation whilst working in the high risk MDR TB environment.’ | 5 |
| Bieh  Port  Harcourt, Nigeria  (2017) | Hospitalized care for MDR-TB in Port  Harcourt, Nigeria: a qualitative study | To (1) explore the physical, social and psychological needs of hospitalized MDR TB patients, (2) examine providers’ perceptions about the hospital based model and (3) discuss the model’s advantages and disadvantages from the patient and the provider perspective. | Focus group discussions and in-depth interviews  Framework  Approach to data analysis. | Not used. | Two focus  group discussions (FGDs) comprising six men and six  women and 11 in-depth interviews (comprising six men  and five women). They selected four healthcare providers  who played key roles in managing MDR-TB patients in the DR TB treatment facility for key informant interviews. | ‘Providers’ fears of infection contributed to stigma and hindered accessibility of care and support services.’ | 8 |
| Brouwer  Manica, Sofala and Tete provinces, Mozambique  (2014) | Healthcare Workers’ Challenges in the Implementation of Tuberculosis Infection Prevention and Control Measures in Mozambique | To investigate Mozambican HCWs’ perceptions of their occupational TB risk and the measures they report using to reduce this risk.  Explore the challenges HCWs encounter while using these TBIPC measures. | Focus group discussions (FGD) in three Mozambican provinces analysed using content method. | Framework for TB infection control implementation (managerial, administrative, environmental, and personal respiratory protection measures) | 86 participants in 11 focus group discussions with participants from all health care facilities in the two provinces (except provincial hospitals). Participants included auxiliary workers, medical staff (doctors, clinical officers and nurses) and TB program  Staff. | ‘HCWs in Mozambique perceive a high occupational risk of TB  infection. They report several challenges using measures to reduce this risk such  as shortage of material, lack of clear guidelines, insufficient motivation and  inadequate training. Robust training with motivational approaches, alongside  supervision and support for HCWs could improve implementation of TBIPC.’ | 8 |
| Buregyeya  Mukono and Wakiso  districts in central Uganda  (2011) | Acceptability of masking and patient separation to control  nosocomial Tuberculosis in Uganda: a qualitative study | The aims of this study were to: (1) explore patients’ opinions about cough etiquette, masking and  patient separation with regard to TB prevention and (2) find out their preferred measures. | In-depth interviews and focus group discussions.  Content analysis was used to analyse data. | None | Eighteen in-depth exit interviews with six patients with possible pulmonary TB  and 12 newly diagnosed pulmonary TB patients. Eight focus group discussions (FGDs) were conducted with health  workers with 58  participants in total. Participants were from all facility levels. | ‘Scaling up effective TB infection control norms and behaviors requires a patient-centered, rights-based, and evidence-based approach. Socially acceptable measures like covering the mouth and nose with a handkerchief should be promoted. We recommend that further studies are needed to explore how community advocacy impacts on acceptability of masking. Furthermore, the efficacy of covering the mouth using a handkerchief or piece of cloth compared to wearing a mask in TB prevention needs to be evaluated.’ | 8 |
| Buregyeya  Mukono and Wakiso  districts in central Uganda  (2013) | Implementation of tuberculosis infection control  in health facilities in Mukono and Wakiso districts,  Uganda | To assess the implementation  of TBIC in health facilities in Mukono and Wakiso districts. In addition, we determined whether facility characteristics were associated with implementation of  TBIC and identified barriers to implementation. | FGDs with the use of an interview guide.  Unclear methods use to analyse data, described as ‘common themes developed’. | None | Eight FGDs were conducted with HCWs at different health facility levels. | ‘Barriers that hamper implementation of TBIC elicited included: under-staffing, lack of space for patient separation, lack of funds to purchase masks, and health workers not appreciating the importance of TBIC.’ | 7 |
| Chapman  San Pedro de Macorís and Santiago provinces in the Dominican Republic.  (2018) | Health care workers’ recommendations for strengthening tuberculosis infection control in the Dominican Republic | To describe HCWs’ practical recommen­dations for improving adherence to M. tuberculosis infection control practices in their health institutions and across other entities in the Dominican Republic. | Focus group discus­sions  Grounded theory and dimen­sional analysis techniques used for data analysis. | No explicit theory | Ten focus group discussions were conducted with 40 participants (24 physicians and 16 nurses) at two tertiary level facilities. | ‘The observed “knowledge–action gap,” described as inconsistencies in HCWs’ application of clinical knowledge to practice (8), results in ineffective TB pre­vention and control in health institutions in the Dominican Republic. Direct con­sultation with front-line HCWs about their perceived limitations of *M. tubercu­losis* infection control practices at the in­stitutional and national levels can add value to their recommendations for enhanced TB control efforts. Likewise, these recommendations can serve as a framework for continued national dia­logue with the Dominican MoH on pol­icy reform.’ | 9 |
| Chapman  Santa Domingo and Santiago province, Dominican Republic  (2017) | Perceived Barriers to Adherence to Tuberculosis Infection Control  Measures among Health Care Workers in the Dominican Republic | To identify, using a qualitative  approach, perceived barriers to adherence to TB infection  control measures among HCWs in the DR. | Individual, semi-structured interviews.  Thematic analysis | Socioecological framework used for study design and data interpretation. | Nine health workers were interviewed at two tertiary-level hospitals. | ‘Ineffective TB infection control strategies coupled with erroneous understanding  or perceptions of TB risk complicate TB management in limited resource  settings. Perceived barriers at the individual or institutional level may hinder how HCWs understand the actual risk of TB transmission and are able to comply with preventive strategies to reduce risk of M. tuberculosis exposure. Addressing such barriers by strengthening infection control program infrastructure and implementing educational interventions within institutions may reduce HCWs’ risk of nosocomial TB transmission.’ | 8 |
| Chapman  Santiago and San Pedro de Macoris province, Dominican Republic  (2017) | The Role of Powerlessness Among Health Care Workers in Tuberculosis Infection  Control | To describe the decision-making process by which  HCWs use preventive strategies to reduce nosocomial M.  tuberculosis transmission in their clinical practice. | Focus group discussions  Grounded theory analysis | Socioecological framework used for study design and data interpretation. Results discussed by referring to social cognitive theory. | Ten focus groups with 40 participants (24 physicians and 16 nurses) at two tertiary level drug-resistant TB institutes. | ‘This study identified how the intrinsic and extrinsic factors influenced the decision-making process among HCWs to adhere to M. tuberculosis infection control measures in two DR health institutions. The knowledge−action gap was observed, influenced primarily by HCWs’ feelings of powerlessness to prevent occupational M. tuberculosis transmission.’ | 10 |
| Cowan  Tigray and Amhara regions in Ethiopia  (2013) | A qualitative assessment of challenges to tuberculosis  management and prevention in Northern Ethiopia | To explore providers’  perspectives regarding barriers to preventing, diagnosing and treating TB in government hospitals  in Tigray and Amhara to identify factors affecting TB  control. | In-depth individual interviews and focus group discussions.  Content analysis and phenomenology. | Not described. | Seventy three study participants: eighteen  individual interviews were conducted and  25 FGDs each involving 2–4 participants at 5 government hospitals. | ‘Subjects shared deep concern regarding the growing perceived prevalence of MDR TB and were acutely aware of their own vulnerability as first-line providers in the absence of appropriate diagnostics and medications, especially in a setting of strained human resources. Providers consistently suggested that their own fear of MDR-TB in a setting with no true access to effective treatment impaired their ability to care for patients.’ | 8 |
| Daftary  KwaZulu Natal province, South Africa  (2016) | Provider perspectives on drug-resistant tuberculosis and  human immunodeficiency virus care in South Africa: a  qualitative case study | To describe a novel qualitative case study examining personal, professional and programmatic factors  influencing health care workers’ (HCWs’) capacity  to deliver optimal care for MDR- and XDR-TB and HIV in the high-burden province of KwaZulu Natal. | Group and individual interviews.  Analysed using grounded theory. | Not described | Five group interviews and  three individual interviews  with 17 health worker participants in total at a tertiary TB facility. | ‘Comprehensive, decentralised management of MDR/XDR-TB and HIV coinfection requires the creation of patient-provider trust and treatment literacy in MDR/XDR-TB programmes, and defying workplace norms that could provoke nosocomial TB exposure and fragmented service provision.’ | 8 |
| Dodor  Sekondi-Takoradi Metropolitan district, Ghana  (2010) | Manifestations of tuberculosis stigma within the healthcare system: The case of Sekondi-Takoradi Metropolitan district in Ghana | To explore the manifestations of tuberculosis (TB) stigma within the healthcare  system. | Individual interviews and focus group discussions analysed using grounded theory principles. | Not described | 21 individual interviews and six focus groups (8 – 12 participants) predominantly health workers working with TB patients at four different health institutions. | ‘Healthcare workers expressed fear of infection when interacting with TB patients; a fear which intensifies after the confirmation of the diagnosis. For fear of infection, they shunned, avoided, and advocated the segregation of TB patients at home and in the hospitals. They sometimes maltreated the patients, and accused and blamed them for deliberately infecting others. Posting to TB units/wards is viewed as a punishment, with majority indicating refusal to work there or be trained as TB nurse/doctor. They maintained that those working at TB units should be given incentives.’ | 8 |
| Fadare  South West Nigeria  (2020) | Nurses’ Safety in Caring for Tuberculosis Patients at a Teaching  Hospital in South West Nigeria | To investigated the challenges faced by  nurses in the care of TB patients in a Federal Teaching  Hospital in Southwest Nigeria. | Individual interviews.  Data analysed using content analysis. | Not described | 20 participants who were professional nurses at a teaching hospital. | ‘The major concern, as expressed by all the nurses, is the fear of getting infected with TB and inadvertently infecting their immediate families, relatives, and other patients. In addition, respondents expressed that senior nurses reassigning cases of TB patients to junior inexperienced officers also is a challenge. Some health workers highlighted the need for a refresher course and retraining on TB, prevention, and current treatment modalities stating that inadequate training is a challenge they face and this study supports this as some nurses identified a need to be trained adequately in managing TB patients.’ | 9 |
| Kallon  Western Cape, South Africa  (2021) | Organisational Culture and Mask-Wearing Practices for  Tuberculosis Infection Prevention and Control among Health  Care Workers in Primary Care Facilities in the Western Cape,  South Africa: A Qualitative Study | To document the ways  that organisational culture might work as a barrier to effective TB-IPC but also to consider  how changes in organisational culture in clinics might support improved IPC in South  Africa and more generally. | Individual interviews and focus group discussions.  Data analysed using hybrid inductive-deductive approach, both thematic networks and framework approach. | Data analysed using Mannion and Davies framework of organisational culture. | Twenty-three individual interviews and four focus group discussions with 25 health workers and facility staff at six primary health care facilities. | ‘Organisational culture—whether manifested in clinic protocols and the practices of HCWs, or more subtly, in the underlying beliefs and narratives that shape how HCWs understand the risks and responsibilities of their work—has an important, and underresearched, impact on HCW mask-wearing and other PPE and IPC practices. | 9 |
| Khaund  Karnataka, India  (2018) | Infection Control Prevention Practices on Pulmonary TB Transmission among Health Care Personnel of Selected Hospital in India | To assess infection control practices that a health care personnel follows to prevent transmission of TB and to evaluate the resources and facilities available in the area and also to identify the factors which helps to improve the practices; thereby to minimize the risk for transmission of pulmonary TB infections. | Focus group discussion.  Thematic analysis | Not described. | One focus group discussion with 14 health workers working in TB units in a tertiary care hospital. | ‘Intermittent interruption of supply (e.g., N95 masks), lack of knowledge, lack of adherence to infection control practices and lack of awareness by the patients were found to be the factors for non-compliance.’ | 4 |
| Kuyinu  Lagos State, Nigeria  (2019) | Tuberculosis infection prevention and control measures in DOTS  centres in Lagos State, Nigeria | To assess the level of implementation of TBIC measures and air exchange rate in DOTS centres in Lagos State. | Focus group discussion.  No method for qualitative data analysis described. | Not described. | Five focus group discussions with 8 – 10 health workers at primary and secondary health facilities with DOTS centres. | ‘The HCWs’ perception of being at risk of contracting TB was reported to affect the way they relate to TB patients. The key barrier to implementing TBIC was the design of DOTS centres.’ | 7 |
| Kuyinu  Lagos State, Nigeria  (2016) | Tuberculosis infection control measures in  health care facilities offering tb services in  Ikeja local government area, Lagos, South  West, Nigeria | To assess the  level of implementation of TBIC measures in all health  facilities caring for TB suspects or cases and to determine  HCWs’ perceptions of barriers to implementation  of TBIC in Ikeja Local Government Area (LGA), Lagos  State, southwest, Nigeria. | Focus group discussions.  No method for qualitative data analysis described. | Not described. | Four focus group discussions with 10 participants each at TB care facilities. | ‘Findings from the focus group discussions showed weak managerial support, poor funding, under-staffing, lack of space and not wanting to be seen as stigmatizing against tuberculosis patients as barriers that hindered the implementation of TB infection control measures.’ | 6 |
| Marais  Western Cape, South Africa  (2019) | Continuity of care for TB patients at a South  African hospital: A qualitative participatory  study of the experiences of hospital staff | To understand factors influencing the inpatient clinical management and discharge of TB patients from the perspectives of staff employed in a public sector acute hospital managed by a Provincial Department of Health. | Focus group discussions with participatory action research approach.  Miles and Huberman framework used for data analysis. | Linked with conceptualizations of patient-centred care. | Eight focus group discussions with a total of 60 health workers at a tertiary hospital. | ‘Nurses expressed a fear of exposure to TB and MDR-TB due to challenges in clinical and infection-prevention control. Clinical hierarchies, poor interdisciplinary teamwork, limited task shifting and poor communication interfered with effective clinical and discharge processes. A high workload, staff shortages and inadequate skills resulted in insufficient information and health education for TB patients and their caregivers. Despite awareness of the patients’ socio-economic challenges, some aspects of care were not patient-centered, and caregivers were not included in discharge planning. Communication between the hospital and referral points was inefficient and poorly supported by information systems.’ | 10 |
| Marme  Madang Province, Papua New Guinea  (2018) | Barriers and facilitators to effective tuberculosis infection control practices in Madang Province, PNG – a  qualitative study. | To investigate nurses’ everyday grassroots experiences of how TBIC measures are implemented in health services in  rural PNG. | Individual and  group semi-structured interviews  Phenomenological hermeneutic approach used for data analysis. | Not described. | 12 nurses in rural healthcare facilities. | ‘With high levels of TB in the community, illness leading people to seek treatment, health services can be TB ‘hot spots’ and, in the absence of good TBIC, a clinical service may actually promote the spread of TB, rather than contain it. It is, therefore, vital that local TBIC policies align with international and national policies and guidelines, to ensure that local practices are consistent with current best practices. Investing in health infrastructure and medical equipment, TBIC training for health employees and community, and adherence towards TBIC measures, may facilitate effective implementation of TBIC at rural health facilities in PNG. Equally importantly, health service managers should address conditions and issues in wider contextual matters that are beyond the abilities of the healthcare workers.’ | 9 |
| Maroldi  São  Paulo, Brazil  (2017) | Adherence to precautions for preventing the transmission of microorganisms in primary health care: a qualitative study | To explore knowledge and barriers to the implementation  of precautions for the prevention of transmission of microorganisms  in PHC, as well as those factors that determine the adherence of professionals to these measures. | Focus group discussions  Content analysis | Not described. | Twenty health professionals participated in four focus groups based at five primary health care units. | ‘To ensure national preparedness to deal with epidemics and pandemics, all health care settings need to ensure a good level of adherence to infection prevention measures. Primary care is (worldwide) the first point of contact in dealing with infectious diseases. These findings show the main issues that should be addressed to improve infection control practice in primary care, to minimize the risk of disease transmission to both patients and health care workers. Raising awareness by promoting knowledge is a key element of clinical practice. Notwithstanding this, to implement guidelines and in-service training, tailored to the local context, is very relevant to clinical practice since PHC is provided in such a variety of environments and situations.’ | 9 |
| Matakanye  Limpopo province, South Africa  (2019) | Caring for Tuberculosis Patients: Understanding the  Plight of Nurses at a Regional Hospital in Limpopo  Province, South Africa | To explore and describe  the experiences of nurses caring for tuberculosis patients at a regional hospital in Vhembe district,  Limpopo Province. | In-depth individual interviews.  Colaizzi’s method used for data analysis. | Not described. | Six nurses based at a regional hospital participated in this study. | ‘Nurses indicated their emotional distress due to fear of contagion, lack of material resources, as well as poor infection control practices. The Occupational Health and Safety Act stipulates measures and standards of practice, however such standards are jeopardized by inadequate financial and resource allocation in the public sector. Nurses expressed the need for in-service education and support by managers. Managers must ensure that nurses have resources to maintain quality nursing care and receive social and psychological support as they face death on a daily basis.’ | 9 |
| Mwenya  Southern Province of Zambia  (2020) | An exploration of health workers risks of contracting tuberculosis in the workplace: a qualitative study | To explore health workers risky behaviors, attitudes and practices that expose them to tuberculosis infection during their professional practice. | Individual, semi-structured interviews.  Thematic analysis. | Not described, but suggests using health belief model in future interventions. | 10 participants, consisting of 1 medical doctor and 9 nurses at a district hospital. | ‘ The risk factors that facilitated tuberculosis transmission from patients to health workers at the hospital included; overcrowding, poor ventilation, absence of an isolation ward, small admission rooms, poor coughing techniques, inadequate personal protective equipment, inadequate tuberculosis infection prevention practices and unhygienic conditions.’ | 9 |
| Nazneen  Multiple hospitals in Bangladesh  (2021) | Implementation status of national  tuberculosis infection control guidelines in  Bangladeshi hospitals | To assess the status of and barriers impeding the implementation of TB IPC measures in  TB specialty hospitals and tertiary care hospitals in Bangladesh. | Individual, open-ended interviews.  Methods described aligns with thematic analysis. | Not described. | 59 participants who were staff at 8 TB specialty hospitals and 3 tertiary care hospitals. | ‘To enable the health system to better implement the national TB IPC guidelines, the guidelines should be introduced in all health settings dealing with TB cases through participatory approaches. Regular, intense monitoring by the NTP’s infection-control coordinating bodies can be maintained as far as triage and segregation of TB patients is concerned. The national TB infection control guidelines should be rolled out in chest-disease hospitals, clinics, and tertiary care hospitals.’ | 10 |
| Padayatchi  Province omitted to protect patient identities, South Africa  (2010) | Case series of the long-term psychosocial impact of  drug- resistant tuberculosis in HIV-negative medical doctors | To present the cases of five HIV-negative doctors in South Africa who were diagnosed with primary drug-resistant TB in 2000–2003 and followed up in 2009, to explore the psychosocial impact of their illness. | Individual, semi-structured interviews.  Content analysis. | Not described. | 5 participants who were medical doctors managed for drug resistant TB at a specialist hospital. | ‘Health care workers are not only at greater risk for acquiring TB, they also serve as potent vectors for TB transmission due to their greater contact with immune-compromised patients. This issue is stronger yet in the cases of MDR-TB and extensively drug-resistant TB and in high HIV prevalence areas. The public sector is already battling against the better salaries and work conditions offered to HCWs in better resourced countries and the private sector.31 There is a clinical and social imperative to prioritise infection control among HCWs, not only to reduce the risk of nosocomial transmission and prolonged morbidity, but also reduce the risk of the ‘brain drain’ in the health workforce.’ | 8 |
| Probandari  Yogyakarta Province, Indonesia  (2019) | Being safe, feeling safe, and stigmatizing attitude among primary health care staff in providing multidrug-resistant tuberculosis care in Bantul District, Yogyakarta Province,  Indonesia | To explore the issue of safety, feeling safe, and stigmatizing attitude among health staff working with MDR-TB cases in PHCs in Bantul district, Yogyakarta Province, Indonesia. | In depth individual interviews.  Analysed using content analysis technique. | Not described. | 22 participants who were clinical staff and TB programme staff 17 primary health care facilities. | ‘Some infrastructures of infection control in PHCs are suboptimal. There was also lacking knowledge of infection control protocols. The stigmatic attitudes manifest in various forms: fear of being infected, avoidance to conduct care, and performing unnecessary overprotected practices of infection control. The cause of the stigmatizing attitude was the fear of being infected. The combination of the suboptimal infection control environment, lacking knowledge, and stigma resulted in unsafe and inefficient practice while doing the MDR-TB care, as well as the discrimination of MDR-TB patients.’ | 8 |
| Sissolak  Western Cape, South Africa.  (2011) | TB infection prevention and control experiences of South African nurses - a phenomenological study | To explore factors influencing TB-IPC practices at hospital level from the experiences of ward nurses in order to identify risks associated with potential nosoco- mial transmission, and to emphasize the crucial role nurses play in TB control and care. | One-to-one semi-structured interviews using phenomenological approach. Content analysed using thematic analysis. | Not described | 20 nurse participants at a tertiary hospital. | ‘Healthcare system inadequacy was the major influence on TB-IPC. IPC provision and practices, TB training for staff and patients, and cross-cultural communication were perceived by nurses to be suboptimal at this large hospital and could increase the risk of nosocomial transmission. The first step should be the implementation and evaluation of a comprehensive contextually appropriate TB-IPC policy with the setting and auditing of standards for appropriate IPC provision and practice across all wards. Concerns and stigma attached to TB should be addressed among both healthcare providers and patients.’ | 10 |
| Tamir  West Gojjam  zone, Northwest Ethiopia  (2016) | Tuberculosis infection control practices and associated factors among health care workers in health centers of West Gojjam zone, Northwest Ethiopia: a cross-sectional study | To determine the level of practice on TBIC and its associated factors among HCWs. | Individual interviews  Thematic coding. | Not described. | 15 participants, each was a key informant at their health centre. | ‘The overall proper TBIC practice by health workers in health centers of West Gojjam Zone was low. The necessary settings and supplies were not available in majority of health centers. Tuberculosis infection control practices of health care workers was determined by their working area/department, knowledge status of TBIC plan and presence of national TBIC guide line in their health centers. Health centers should prepare TBIC plans, orient all health workers and should monitor the activity accordingly.’ | 5 |
| Tshitangano  Limpopo, South Africa  (2014) | The practices of isolating tuberculosis infectious patients at hospitals of Vhembe district, Limpopo Province | This article aims to describe the use of masks and isolation of infectious TB patients at hospitals of Vhembe district, Limpopo Province in order to inform future policy and practices. | Focus group discussions, data analysed using open coding method following Tesch’s 8-step criteria | Not described | 7 focus groups with 57 health workers at 7 rural hospitals. | ‘This study discovered that TB inpatients were not isolated and that the TB cubicles were not reserved for patients with infectious TB. Furthermore, the movement of TB inpatients in isolation was not restricted and masks were not used either consistently or appropriately by patients, staff or visitors in wards that cared for infectious TB patients.’ | 8 |
| Tshitangano  Limpopo, South Africa  (2013) | Availability of tuberculosis infection control plans at rural hospitals of Vhembe district, Limpopo Province  of South Africa | To investigate the availability of TB IC plans at the rural hospital of Vhembe district in order to:  - explore and describe the awareness of health care providers regarding the availability of TB IC in the hospital  - assess the knowledge of health care providers on the content of the TB IC plan  - identify the role of infection control committees from the perspective of health care providers. | Focus group discussions.  Tesch’s eight–step open-coding method. | Not described | 7 focus groups with 57 health workers at 7 rural hospitals. | ‘HCWs were not aware of the availability and the information contained in the TB IC plans. No person was designated as TB IC officer at hospital level. There was lack of a TB IC Committee and teams as well as ineffective utilisation of those that did exist.’ | 7 |
| Tshitangano  Limpopo, South Africa  (2014) | Measures practised by healthcare workers to prevent tuberculosis transmission at rural hospitals in Vhembe district | To explore and describe the following action taken by HCWs:  • Screening measures.  • Education measures.  • Separation measures.  • Triage measures.  • Tuberculosis investigation tests. | Focus group discussions.  Tesch’s eight-step, open-coding method | Not described | Seven focus groups with 57 health workers at seven rural hospitals. | ‘Analysis of measures practised by HCWS at rural hospitals of Vhembe district revealed that there is delayed tuberculosis suspicion at rural hospitals in Vhembe district, aggravated by failure to assign responsibility to personnel to immediately screen patients who cough for a prolonged duration upon arrival at the facility. Failing to designate anybody in this regard was seen as a contributing factor to tissues not being provided to tuberculosis suspects, instructions on coughing hygiene not being offered to identified tuberculosis suspects, masks not being handed out, and tuberculosis suspects not being separated from other patients, and their treatment not being accelerated. The impact of a lack of a designated person was seen by the lack of uniformity with regard to the time taken to collect the first sputum specimen for tuberculosis diagnosis. This leads to delayed tuberculosis investigation and tuberculosis diagnosis.’ | 8 |
| Tudor  Multiple provinces in South Africa  (2013) | Health care workers’ fears associated with working in multidrug- and or extensively-resistant tuberculosis wards in South Africa | To assess health care workers’ (HCWs’) fears of working in multidrug-resistant TB (MDR-TB) or extensively drug-resistant TB (XDR-TB) wards. | Open-ended question in questionnaire.  Data analysed using content analysis | Not described | 499 HCWs based at drug-resistant TB hospitals participated in the study, with 363 (73%) responding to the open-ended question. | ‘This study highlights the fears of HCWs across South Africa working in MDR-/XDR-TB wards. HCWs reported the fear of developing MDR-/XDR-TB and concerns over the work environment, both of which may prevent HCWs from providing patients with the best care possible. This analysis suggests that many of the most common reasons for fear can be addressed through basic educational, administrative and environmental interventions. It is imperative that efforts are made to improve IC and ensure safe working conditions for all HCWs.’ | 8 |
| Van der Westhuizen  Western Cape,  South Africa  (2017) | When students become patients: TB disease among medical undergraduates in Cape Town, South Africa | To: (i) investigate  the clinical presentation, diagnostic investigations and treatment of TB in medical students; (ii) describe students’ experiences of developing occupational TB; and (iii) evaluate the support systems utilised following TB diagnosis. | Semi-structured individual interviews (and open responses to questionnaire.  Framework approach used to analyse data. | Not described. | Twelve medical students based at two universities participated in four in-depth interviews. | ‘Students reported poor implementation of TB-IC in their training institutions as their major risk factor for occupational disease.’ | 8 |
| Woith  Faciliaties in two regions of Russia.  (2012) | Barriers and Facilitators Affecting Tuberculosis Infection Control Practices of Russian Health Care Workers | To identify barriers and motivators to use of IC measures among Russian HCWs. | Focus group discussions.  Data analysed by ‘ coding clustered responses into themes.’ | Recommends the use of Social Cognitive Theory or Collectivist Theory for future interventions.. | 96 participants from three TB hospitals and two out-patient TB clinics. Participants included physicians, nurses, laboratory staff and support staff. | ‘Our results highlight the need for evaluation of current educational programs, and TB program administrators in these regions might consider implementing curricula based on social cognitive theory, incorporating role models and mentoring. Positive health messages, which have been successful  in promoting health-related behavior change elsewhere, might be successful in promoting the use of TB IC. Wise investment of limited resources is  necessary to make the most of available funds, and a creative evaluation of budgets could result in the elimination of unnecessary expenses; however, administrators might also consider the impact of resource redistribution on employee satisfaction. Finally, individualized rewards based on personal motivators or group rewards that build on collectivist theory could also be explored.’ | 8 |
| Zelnick  KwaZulu Natal, South Africa  (2013) | Health Care Worker Perspectives on Workplace Safety, Infection Control and Drug-Resistant Tuberculosis in a High Burden HIV setting | To use frontline perspectives to identify challenges that HCWs face implementing measures to reduce TB risk, and to expand our understanding of how to improve workplace safety. | FGD and questionnaire with same questions as focus groups.  Analysing data using constant comparative method. | Not described. | 19 focus group discussions involving 55 hospital staff and 7 paper based interviews with hospital managers and IC personnel. | ‘HCWs expressed confusion over inconsistencies between policies and their implementation: Always wear the N95 mask, or conserve them due to expense? Separate XDR and MDR TB patients, or allow them to mix at times? Contradictions between policy and practice lead to seeming distrust of policies among HCWs.  HCWs in our study described using their own discretion in deciding when to wear N95 masks and open windows.’ | 9 |
| Zinatsa  Free State, South Africa  (2018) | Voices from the frontline: barriers and strategies to improve tuberculosis infection control in primary health care facilities in South Africa | This study sought to 1) identify factors influencing TB infection control behaviour at PHC level within a high TB burden district and 2) in a participatory manner elicit recommendations from HCWs for improved TB infection control. | Focus group discussions.  Data analysed using Braun and Clark’s thematic analysis method. | Information, motivation and behaviour model used to design study and interpret findings. Study used a participatory approach to research. | 5 focus group discussions, 7 – 10 participants each, 55 participants in total based at primary health care facilities. The participants were TB nurses and facility managers. | ‘TB infection control remains problematic in PHC facilities. HCWs need to change their behaviours and adhere to TB infection control guidelines. Behaviour change is not an easy once off event, it is a process that requires time and motivation. This study followed a participatory approach, which allowed HCWs to identify and prioritise strategies to improve TB infection control behaviours and included: training for comprehensive TB infection control for all HCWs; clarity on TB infection control policy guidelines; improved patient education and awareness of TB infection control measures; emphasis on the active role HCWs can play in infection control as change agents; improved social support; practical, hands-on training or role playing to improve behavioural skills; and the destigmatisation of TB/HIV among HCWs and patients.’ | 10 |
