# Supplementary material for: Health worker experiences of implementing TB infection prevention and control: A qualitative evidence synthesis to inform implementation recommendations
Source: PLOS Glob Public Health. 2022 Jul 7;2(7):e0000292. doi: 10.1371/journal.pgph.0000292 (PMC10021216; doi:10.1371/journal.pgph.0000292)
Supplement: S2 Table — (DOCX) [file pgph.0000292.s004.docx]

| **S3 Table:**  **Critical Appraisals Skills Programme (CASP) scores of included studies** | | | | | | | | | | | | |
| --- | --- | --- | --- | --- | --- | --- | --- | --- | --- | --- | --- | --- |
| **Lead author**  **Country where study was based**  **(Year)** | **Title of Publication** | **Q1** | **Q2** | **Q3** | **Q4** | **Q5** | **Q6** | **Q7** | **Q8** | **Q9** | **Q10** | **CASP score**  **(out of 10)** |
| Adeleke  Khayelitsha, Western Cape, South Africa  (2012) | Barriers to implementation of Tuberculosis infection control amongst South African health care workers. | Yes | Yes | Yes | Can’t tell | Can’t tell | No | No | Can’t tell | No | No | 3 |
| Adu  Gauteng, Western Cape and KwaZulu-Natal provinces of South Africa  (2020) | Perceived Health System Barriers to Tuberculosis Control Among Health Workers in South Africa | Yes | Yes | Yes | Yes | Yes | No | Yes | Yes | Yes | Yes | 9 |
| Akshaya  Karnataka,  India  (2017) | “Who has to do it at the end of the day?  Programme officials or hospital authorities?”  Airborne infection control at drug resistant  tuberculosis (DR-TB) centres of Karnataka,  India: a mixed-methods study | Yes | Yes | Can’t tell | No | No | No | Yes | Yes | No | No | 4 |
| Arjun  KwaZulu Natal, South Africa  (2013) | Enrolled nurses' experiences of caring for multi drug resistant tuberculosis patients in the KwaZulu Natal province of South Africa | Yes | Yes | Yes | No | Yes | No | Yes | No | No | No | 5 |
| Bieh  Port  Harcourt, Nigeria  (2017) | Hospitalized care for MDR-TB in Port  Harcourt, Nigeria: a qualitative study | Yes | Yes | Yes | Yes | Yes | No | Yes | Yes | Can’t tell | Yes | 8 |
| Brouwer  Manica, Sofala and Tete provinces, Mozambique  (2014) | Healthcare Workers’ Challenges in the Implementation of Tuberculosis Infection Prevention and Control Measures in Mozambique | Yes | Yes | Yes | Yes | Yes | No | Yes | Yes | Can’t tell | Yes | 8 |
| Buregyeya  Mukono and Wakiso  districts in central Uganda  (2011) | Acceptability of masking and patient separation to control  nosocomial Tuberculosis in Uganda: a qualitative study | Yes | Yes | Yes | Yes | Yes | No | Yes | Can’t tell | Yes | Yes | 8 |
| Buregyeya  Mukono and Wakiso  districts in central Uganda  (2013) | Implementation of tuberculosis infection control  in health facilities in Mukono and Wakiso districts,  Uganda | Yes | Yes | Yes | Can’t tell | Yes | No | Yes | Can’t tell | Yes | Yes | 7 |
| Chapman  San Pedro de Macorís and Santiago provinces in the Dominican Republic.  (2018) | Health care workers’ recommendations for strengthening tuberculosis infection control in the Dominican Republic | Yes | Yes | Yes | Yes | Yes | No | Yes | Yes | Yes | Yes | 9 |
| Chapman  Santa Domingo and Santiago province, Dominican Republic  (2017) | Perceived Barriers to Adherence to Tuberculosis Infection Control  Measures among Health Care Workers in the Dominican Republic | Yes | Yes | Yes | No | Can’t tell | Yes | Yes | Yes | Yes | Yes | 8 |
| Chapman  Santiago and San Pedro de Macoris province, Dominican Republic  (2017) | The Role of Powerlessness Among Health Care Workers in Tuberculosis Infection  Control | Yes | Yes | Yes | Yes | Yes | Yes | Yes | Yes | Yes | Yes | 10 |
| Cowan  Tigray and Amhara regions in Ethiopia  (2013) | A qualitative assessment of challenges to tuberculosis  management and prevention in Northern Ethiopia | Yes | Yes | Yes | Yes | Yes | No | Yes | Yes | Yes | No | 8 |
| Daftary  KwaZulu Natal province, South Africa  (2016) | Provider perspectives on drug-resistant tuberculosis and  human immunodeficiency virus care in South Africa: a  qualitative case study | Yes | Yes | Yes | Yes | Yes | No | Yes | Yes | Yes | No | 8 |
| Dodor  Sekondi-Takoradi Metropolitan district, Ghana  (2010) | Manifestations of tuberculosis stigma within the healthcare system: The case of Sekondi-Takoradi Metropolitan district in Ghana | Yes | Yes | Yes | Yes | Yes | No | Can’t tell | Yes | Yes | Yes | 8 |
| Fadare  South West Nigeria  (2020) | Nurses’ Safety in Caring for Tuberculosis Patients at a Teaching  Hospital in South West Nigeria | Yes | Yes | Yes | Yes | Yes | No | Yes | Yes | Yes | Yes | 9 |
| Kallon  Western Cape, South Africa  (2021) | Organisational Culture and Mask-Wearing Practices for  Tuberculosis Infection Prevention and Control among Health  Care Workers in Primary Care Facilities in theWestern Cape,  South Africa: A Qualitative Study | Yes | Yes | Yes | Yes | Yes | No | Yes | Yes | Yes | Yes | 9 |
| Khaund  Karnataka, India  (2018) | Infection Control Prevention Practices on Pulmonary TB Transmission among Health Care Personnel of Selected Hospital in India | Yes | Yes | Can’t tell | No | Yes | No | Yes | No | No | No | 4 |
| Kuyinu  Lagos State, Nigeria  (2019) | Tuberculosis infection prevention and control measures in DOTS  centres in Lagos State, Nigeria | Yes | Yes | Yes | No | Yes | No | Yes | No | Yes | Yes | 7 |
| Kuyinu  Lagos State, Nigeria  (2016) | Tuberculosis infection control measures in  health care facilities offering tb services in  Ikeja local government area, Lagos, South  West, Nigeria | Yes | Yes | Yes | No | Yes | No | Yes | No | Yes | No | 6 |
| Marais  Western Cape, South Africa  (2019) | Continuity of care for TB patients at a South  African hospital: A qualitative participatory  study of the experiences of hospital staff | Yes | Yes | Yes | Yes | Yes | Yes | Yes | Yes | Yes | Yes | 10 |
| Marme  Madang Province, Papua New Guinea  (2018) | Barriers and facilitators to effective tuberculosis infection control practices in Madang Province, PNG – a  qualitative study. | Yes | Yes | Yes | Yes | Yes | No | Yes | Yes | Yes | Yes | 9 |
| Maroldi  São  Paulo, Brazil  (2017) | Adherence to precautions for preventing the transmission of microorganisms in primary health care: a qualitative study | Yes | Yes | Yes | Yes | Yes | No | Yes | Yes | Yes | Yes | 9 |
| Matakanye  Limpopo province, South Africa  (2019) | Caring for Tuberculosis Patients: Understanding the  Plight of Nurses at a Regional Hospital in Limpopo  Province, South Africa | Yes | Yes | Yes | Yes | Yes | No | Yes | Yes | Yes | Yes | 9 |
| Mwenya  Southern Province of Zambia  (2020) | An exploration of health workers risks of contracting tuberculosis in the workplace: a qualitative study | Yes | Yes | Yes | Yes | Yes | No | Yes | Yes | Yes | Yes | 9 |
| Nazneen  Multiple hospitals in Bangladesh  (2021) | Implementation status of national  tuberculosis infection control guidelines in  Bangladeshi hospitals | Yes | Yes | Yes | Yes | Yes | Yes | Yes | Yes | Yes | Yes | 10 |
| Padayatchi  Province omitted to protect patient identities, South Africa  (2010) | Case series of the long-term psychosocial impact of  drug- resistant tuberculosis in HIV-negative medical doctors | Yes | Yes | Can’t tell | Yes | Yes | No | Yes | Yes | Yes | Yes | 8 |
| Probandari  Yogyakarta Province, Indonesia  (2019) | Being safe, feeling safe, and stigmatizing attitude among primary health care staff in providing multidrug-resistant tuberculosis care in Bantul District, Yogyakarta Province,  Indonesia | Yes | Yes | Yes | Yes | Can’t tell | No | Yes | Yes | Yes | Yes | 8 |
| Sissolak  Western Cape, South Africa.  (2011) | TB infection prevention and control experiences of South African nurses - a phenomenological study | Yes | Yes | Yes | Yes | Yes | Yes | Yes | Yes | Yes | Yes | 10 |
| Tamir  West Gojjam  zone, Northwest Ethiopia  (2016) | Tuberculosis infection control practices and associated factors among health care workers in health centers of West Gojjam zone, Northwest Ethiopia: a cross-sectional study | Yes | Yes | Yes | Can’t tell | Can’t tell | No | Yes | Can’t tell | No | Yes | 5 |
| Tshitangano  Limpopo, South Africa  (2014) | The practices of isolating tuberculosis infectious patients at hospitals of Vhembe district, Limpopo Province | Yes | Yes | Yes | Yes | Yes | No | Yes | Yes | Yes | No | 8 |
| Tshitangano  Limpopo, South Africa  (2013) | Availability of tuberculosis infection control plans at rural hospitals of Vhembe district, Limpopo Province  of South Africa | Yes | Yes | Yes | Can’t tell | Can’t tell | No | Yes | Yes | Yes | Yes | 7 |
| Tshitangano  Limpopo, South Africa  (2014) | Measures practised by healthcare workers to prevent tuberculosis transmission at rural hospitals in Vhembe district | Yes | Yes | Yes | Yes | Yes | No | Yes | Yes | Yes | No | 8 |
| Tudor  Multiple provinces in South Africa  (2013) | Health care workers’ fears associated with working in multidrug- and or extensively-resistant tuberculosis wards in South Africa | Yes | Yes | Yes | Yes | Yes | No | Yes | Yes | Yes | No | 8 |
| Van der Westhuizen  Western Cape,  South Africa  (2017) | When students become patients: TB disease among medical undergraduates in Cape Town, South Africa | Yes | Yes | Yes | Yes | Can’t tell | No | Yes | Yes | Yes | Yes | 8 |
| Woith  Faciliaties in two regions of Russia.  (2012) | Barriers and Facilitators Affecting Tuberculosis Infection Control Practices of Russian Health Care Workers | Yes | Yes | Yes | Yes | Yes | No | Yes | No | Yes | Yes | 8 |
| Zelnick  KwaZulu Natal, South Africa  (2013) | Health Care Worker Perspectives on Workplace Safety, Infection Control and Drug-Resistant Tuberculosis in a High Burden HIV setting | Yes | Yes | Yes | Yes | Yes | No | Yes | Yes | Yes | Yes | 9 |
| Zinatsa  Free State, South Africa  (2018) | Voices from the frontline: barriers and strategies to improve tuberculosis infection control in primary health care facilities in South Africa | Yes | Yes | Yes | Yes | Yes | Yes | Yes | Yes | Yes | Yes | 10 |
